# Refining Susceptibility Loci of Chronic Obstructive Pulmonary Disease with Lung eqtls

**DOI:** 10.1371/journal.pone.0070220

**Published:** 2013-07-30

**Authors:** Maxime Lamontagne, Christian Couture, Dirkje S. Postma, Wim Timens, Don D. Sin, Peter D. Paré, James C. Hogg, David Nickle, Michel Laviolette, Yohan Bossé

**Affiliations:** 1 Centre de recherche de l’Institut universitaire de cardiologie et de pneumologie de Québec, Québec, Canada; 2 University of Groningen, University Medical Center Groningen, Department of Pulmonology, GRIAC Research Institute, Groningen, The Netherlands; 3 University of Groningen, University Medical Center Groningen, Department of Pathology and Medical Biology, GRIAC Research Institute, Groningen, The Netherlands; 4 University of British Columbia James Hogg Research Center, Center for Heart and Lung Health, St. Paul’s Hospital, Vancouver, British Columbia, Canada; 5 Respiratory Division, Department of Medicine, The University of British Columbia, Vancouver, British Columbia, Canada; 6 Department of Pathology and Laboratory Medicine, The University of British Columbia, Vancouver, British Columbia, Canada; 7 Merck & Co. Inc., Rahway, New Jersey, United States of America; 8 Department of Molecular Medicine, Laval University, Québec, Canada; MOE Key Laboratory of Environment and Health, School of Public Health, Tongji Medical College, Huazhong University of Science and Technology, China

## Abstract

Chronic obstructive pulmonary disease (COPD) is the fourth leading cause of mortality worldwide. Recent genome-wide association studies (GWAS) have identified robust susceptibility loci associated with COPD. However, the mechanisms mediating the risk conferred by these loci remain to be found. The goal of this study was to identify causal genes/variants within susceptibility loci associated with COPD. In the discovery cohort, genome-wide gene expression profiles of 500 non-tumor lung specimens were obtained from patients undergoing lung surgery. Blood-DNA from the same patients were genotyped for 1,2 million SNPs. Following genotyping and gene expression quality control filters, 409 samples were analyzed. Lung expression quantitative trait loci (eQTLs) were identified and overlaid onto three COPD susceptibility loci derived from GWAS; 4q31 (*HHIP*), 4q22 (*FAM13A*), and 19q13 (*RAB4B*, *EGLN2*, *MIA*, *CYP2A6*). Significant eQTLs were replicated in two independent datasets (n = 363 and 339). SNPs previously associated with COPD and lung function on 4q31 (rs1828591, rs13118928) were associated with the mRNA expression of *HHIP*. An association between mRNA expression level of *FAM13A* and SNP rs2045517 was detected at 4q22, but did not reach statistical significance. At 19q13, significant eQTLs were detected with *EGLN2*. In summary, this study supports *HHIP*, *FAM13A*, and *EGLN2* as the most likely causal COPD genes on 4q31, 4q22, and 19q13, respectively. Strong lung eQTL SNPs identified in this study will need to be tested for association with COPD in case-control studies. Further functional studies will also be needed to understand the role of genes regulated by disease-related variants in COPD.

## Introduction

Chronic obstructive pulmonary disease (COPD) is the fourth most common cause of death worldwide and is predicted to be the third leading cause of mortality in the world by the year 2030 [1]. COPD is a complex disease characterized by airflow obstruction that is not fully reversible [2]. Cigarette smoking is the most important cause of the rapid decline in pulmonary function that leads to COPD, but not all smokers develop the disease [3]. Moreover, there is familial aggregation of COPD suggesting a genetic contribution [4]. The only well-established genetic risk factors are inherited mutations causing α1-antitrypsin deficiency [5]. However, these mutations occur in only 1–5% of COPD patients [6].

The number of susceptibility genes for COPD is expanding rapidly with lists tabulated at 57 genes in 2009 [7] and at 144 genes in 2012 [8]. Recent genome-wide association studies (GWAS) have identified four susceptibility loci associated with COPD including 4q22 (*FAM13A*), 4q31 (*HHIP*), 15q25 (*CHRNA3*/*CHRNA5*/*IREB2*) and 19q13 (*RAB4B*, *EGLN2*, *MIA*, *CYP2A6*) [9]–[11]. The causal genes and genetic mechanisms mediating the risk within these loci remain to be found.

The goal of this study is to identify lung expression quantitative trait loci (eQTL) within COPD susceptibility loci identified by GWAS. As part of the lung eQTL consortium, we have recently performed a genome-wide search for eQTLs in 1,111 human lung samples [12]. A predefined hypothesis of the consortium was that human lung eQTLs will identify the most informative markers and improve the localization of causal variants/genes in GWAS-nominated COPD loci [13].

## Methods

### Ethics Statement

All lung tissue samples were obtained in accordance with Institutional Review Board guidelines at the three sites: Laval University (Quebec, Canada), University of British-Columbia (Vancouver, Canada) and Groningen University (Groningen, The Netherlands). All patients provided written informed consent and the study was approved by the ethics committees of the Institut universitaire de cardiologie et de pneumologie de Québec (IUCPQ) and the UBC-Providence Health Care Research Institute Ethics Board for Laval and UBC, respectively. The study protocol was consistent with the Research Code of the University Medical Center Groningen and Dutch national ethical and professional guidelines (“Code of conduct; Dutch federation of biomedical scientific societies”; http://www.federa.org).

### Study Subjects and Lung Specimens

Study subjects and lung specimens were described previously [12], [14]. Briefly subjects were from three sites, Laval University, University of British Columbia, and University of Groningen (henceforth referred to as Laval, UBC, and Groningen, respectively). At Laval, the lung specimens were provided by the IUCPQ site of the Respiratory Health Network Tissue Bank of the Fonds de recherche du Québec – Santé (FRQS) (www.tissuebank.ca); at Groningen, the lung specimens were provided by the local tissue bank of the Department of Pathology, and at UBC, the lung specimens were provided by the James Hogg Research Center Biobank at St Paul's Hospital. COPD diagnosis and severity were determined according to the GOLD recommendations [2]. Clinical characteristics of subjects by site are shown in Table 1.

**Table 1:** Clinical characteristics of patients that passed gene expression and genotyping quality control filters.

	Laval (n = 409)	UBC (n = 339)	Groningen (n = 363)
Male (%)	55.9	53.7	53.2
Age (years)	63.3±9.9	60.2±14.3	51.5±15.5 [9]
Body Mass Index (kg/m^2^)	26.7±5.3	25.6±5.4 [56]	23.2±4.2 [42]
FEV_1_ predicted - pre-BD* (%)	80.5±18.9 [16]	78.2±24.4 [77]	60.5±30.0 [194]
FVC predicted – pre-BD* (%)	89.8±16.1 [31]	86.9±20.1 [75]	75.0±26.5 [208]
FEV1/FVC	0.67±0.10 [32]	0.67±0.13 [77]	0.64±0.19 [189]
COPD	211 (51.6%) [34]	115 (33.9%) [99]	158 (43.5%) [120]
Stage 1 : Mild	82 (38.9%)	43 (37.4%)	20 (12.6%)
Stage 2 : Moderate	117 (55.4%)	60 (52.2%)	38 (23.9%)
Stage 3 : Severe	11 (5.2%)	2 (1.7%)	21 (13.2%)
Stage 4 : Very Severe	1 (0.5%)	10 (8.7%)	69 (43.4%)
Asthma	15 (3.7%)	22 (6.5%)	0 (0.0%)
Diabetes	41 (10.0%)	13 (3.8%)	27 (7.4%)
Cardiac diseases	120 (29.3%)	46 (13.6%)	28 (7.7%)
Smoking			
Smoker	90 (22.0%)	98 (28.9%)	57 (15.7%)
Ex-Smoker	283 (69.2%)	163 (48.1%)	185 (51.0%)
Non-Smoker	36 (8.8%)	26 (7.7%)	100 (27.5%)
Not available	0 (0.0%)	52 (15.3%)	21 (5.8%)
Pack-years in ever-smokers	48.5±27.5 [37]	44.7±28.5 [58]	31.2±17.4 [51]

FEV_1_ : forced expiratory volume in 1 second.

FVC : forced vital capacity.

[-] = missing value.

* pre-BD: pre-bronchodilator.

### Assays

Genome-wide gene expression and genotyping profiles were obtained using a custom Affymetrix array (see GEO platform GPL10379) and the Illumina Human1M-Duo BeadChip array, respectively. Gene expression data are available through the Gene Expression Omnibus (GEO) repository with the accession number GSE23546. Standard quality controls were performed as described previously and only subjects that passed genotyping and expression quality controls were included in this study with 409, 363, and 339 subjects from Laval, Groningen, and UBC, respectively [12].

### COPD Susceptibility Loci

Lung eQTLs were overlaid onto COPD susceptibility loci identified by previous GWAS except for the 15q25-*CHRNA3*/*CHRNA5*/*IREB2* locus that we have reported on previously [15]. Three COPD loci were considered; 4q22 (*FAM13A*), 4q31 (*HHIP*) and 19q13 (*RAB4B*, *EGLN2*, *MIA*, *CYP2A6*). SNPs associated with COPD from previous GWAS were tabulated for the three loci (Table 2). SNPs genotyped in the lung eQTL consortium located 1 Mb up and downstream of the most distant associated SNPs in both directions were evaluated. Chromosomes 4q22 (88,875,909-90,886,297), 4q31 (144,480,780-146,506,456) and 19q13 (40,292,404-42,302,706) include 718, 412 and 739 SNPs, respectively. Genes residing in the same regions were tested as *cis*-eQTLs for probe sets for 14 genes on 4q22 (*SPP1*, *PKD2*, *ABCG2*, *PPM1K*, *HERC6*, *HERC5*, *PIGY*, *HERC3*, *NAP1L5*, *FAM13A*, *TIGD2*, *GPRIN3*, *SNCA*, *MMRN1*), 9 genes on 4q31 (*FREM3*, *GYPE*, *GYPB*, *GYPA*, *HHIP*, *ANAPC10*, *ABCE1*, *OTUD4*, *SMAD1*) and 45 genes on 19q13 (*DYRK1B*, *FBL*, *FCGBP*, *PSMC4*, *ZNF546*, *ZNF780B*, *ZNF780A*, *MAP3K10*, *TTC9B*, *CNTD2*, *AKT2*, *C19orf47*, *PLD3*, *PRX*, *SERTAD1*, *SERTAD3*, *BLVRB*, *SPTBN4*, *SHKBP1*, *LTBP4*, *NUMBL*, *ADCK4*, *ITPKC*, *C19orf54*, *SNRPA*, *EGLN2*, *CYP2G1P*, *CYP2B6*, *CYP2A13*, *CYP2S1*, *AXL*, *HNRNPUL1*, *TGFB1*, *B9D2*, *TMEM91*, *EXOSC5*, *BCKDHA*, *B3GNT8*, *ATP5SL*, *LOC100505495*, *CEACAM21*, *CEACAM4*, *CEACAM7*, *CEACAM5*, *CEACAM6*).

**Table 2:** SNPs associated with COPD in previous GWAS.

Locus	SNP	SNP position	Study
4q22	rs1964516	89,875,909	Cho et al. 2012. Human Molecular Genetics.^11^
	rs7671167	89,883,979	Cho et al. 2010. Nature Genetics.^10^
			Cho et al. 2012. Human Molecular Genetics.^11^
	rs1903003	89,886,297	Cho et al. 2010. Nature Genetics.^10^
4q31	rs1828591	145,480,780	Cho et al. 2010. Nature Genetics.^10^
			Pillai et al. 2009. PLoS Genetics.^9^
	rs13118928	145,486,389	Cho et al. 2012. Human Molecular Genetics.^11^
			Pillai et al. 2009. PLoS Genetics.^9^
	rs13141641	145,506,456	Cho et al. 2012. Human Molecular Genetics.^11^
19q13	rs2604894	41,292,404	Cho et al. 2012. Human Molecular Genetics.^11^
	rs7937	41,302,706	Cho et al. 2012. Human Molecular Genetics.^11^

### Statistical Analysis

Lung eQTLs were identified using a different model than in our previous genome-wide lung eQTL mapping study [12]. Expression data were adjusted for age, sex, and smoking status using robust residuals obtained with the robust fitting of linear models function (rlm) in the R statistical package MASS. Residuals values deviating from the median by more than three standard deviations were filtered as outliers. Association tests between adjusted expression traits and SNPs were performed using quantitative association tests implemented in PLINK [16] (version 1.07). Association tests were performed with the “assoc” command and the Wald test asymptotic p-values were used. Each possible combination of SNPs and genes were tested in the three COPD susceptibility loci in the Laval dataset. Significant *cis*-eQTLs were those passing Bonferroni correction considering the effective number of independent SNPs and genes tested at each locus. The number of independent variables was determined by using the definition of Li and Ji [17], as implemented in SNPSpD [18]. P value thresholds were set at 5.10×10^−6^ for 4q22 (0.05/(279.64 SNPs×35 probesets), 1.50×10^−5^ for 4q31 (0.05/(128.26 SNPs×26 probesets) and 3.43×10^−6^ for 19q13 (0.05/(246.73 SNPs×59 probesets). Significant eQTLs in Laval dataset were then validated in the UBC and Groningen datasets. P values lower than 0.05 were considered significant in the replication sets.

## Results

### Lung eQTLs in the 4q22 Locus

**Figure 1:**
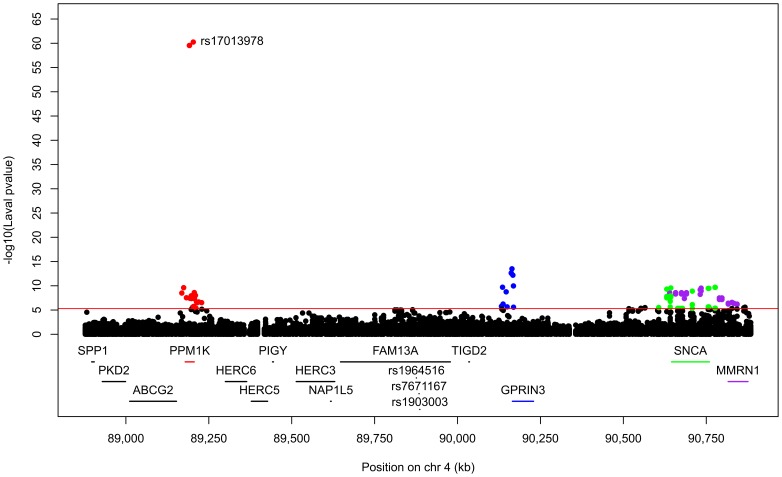
Lung eQTLs on 4q22 in the Laval dataset. Each dot represents an association test between one SNP and one probeset. Only dots above the red line are significant (p<5.10×10^−6^). Significant SNPs were regulating the expression levels of *PPM1K* in red, *GPRIN3* in blue, *SNCA* in green and *MMRN1* in purple. The SNP with the smaller p-value is indicated. SNPs previously associated with COPD are presented at the bottom.

**Figure 2:**
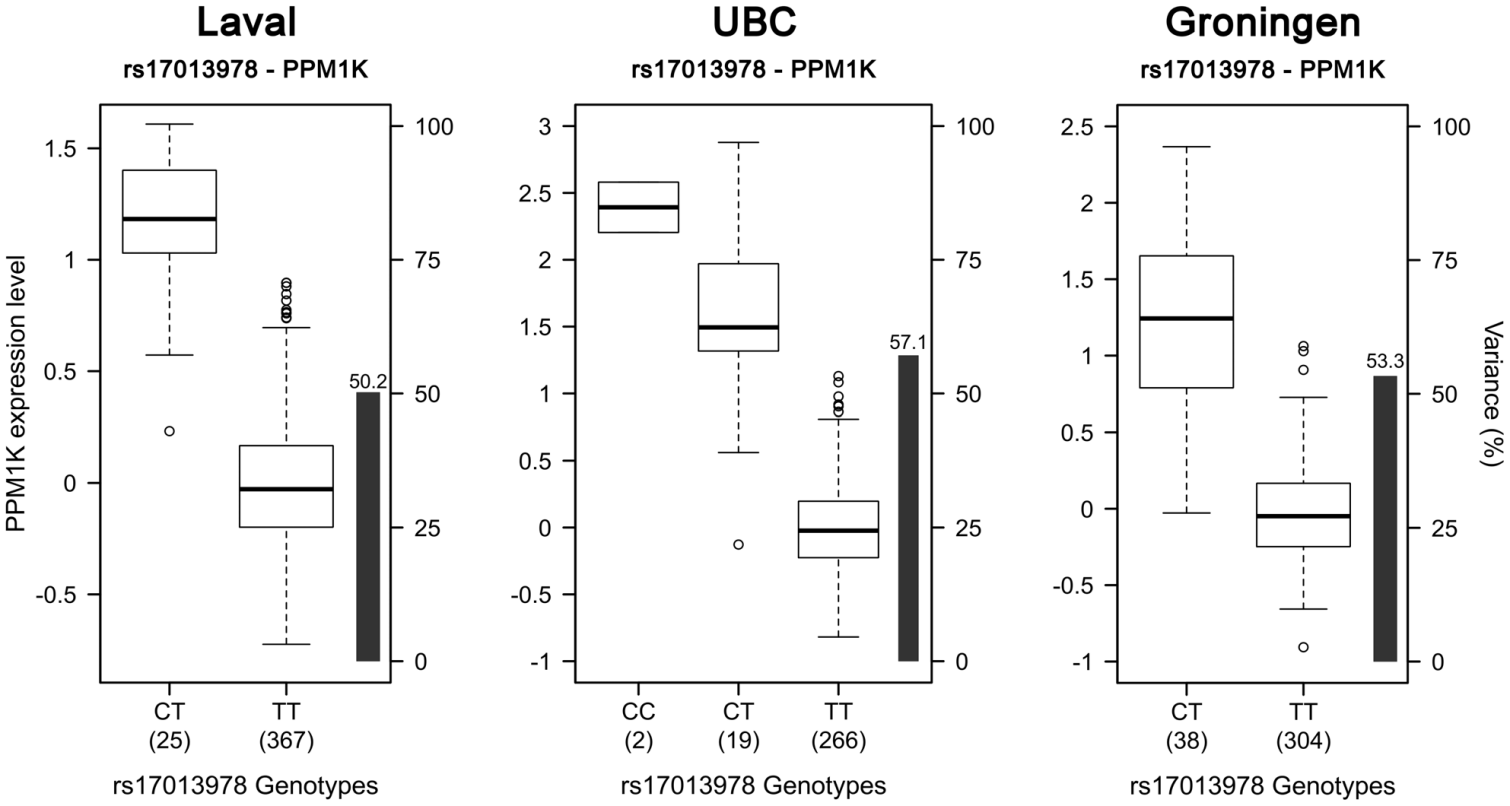
Boxplots of gene expression levels in the lung for PPM1K according to genotype groups for SNP rs17013978. The left y-axis shows the mRNA expression levels for *PPM1K*. The x-axis represents the three genotyped groups for SNP rs17013978. The right y-axis shows the proportion of the gene expression variance explained by the SNP (black bar). Each panel represents a different cohort: Laval (n = 392), UBC (n = 287), Groningen (n = 342). The eQTL p-values were 5.6×10^−61^, 2.8×10^−51^ and 3.8×10^−55^, respectively.

**Figure 3:**
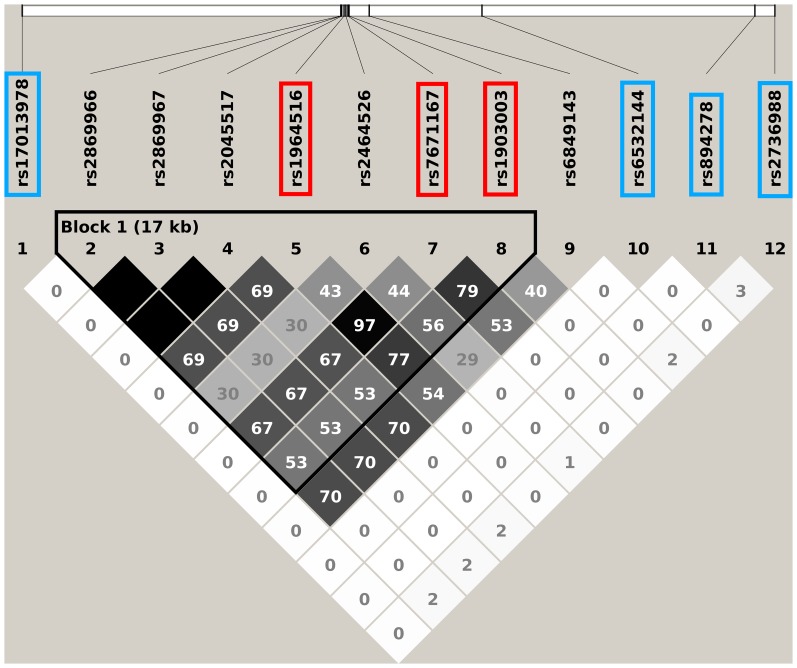
Linkage disequilibrium plot of selected SNPs on the 4q22 locus in the 1000 Genome Project. The white horizontal bar on the upper part of the figure illustrates the location of SNPs on a physical scale. LD values (r^2^) are indicated in each box. The color of the squares illustrates the strength of pairwise r^2^ values on a black and white scale where black indicates perfect LD (r^2^ = 1) and white indicates perfect equilibrium (r^2^ = 0). The genotypes are from the 1000 Genome Project interim phase 1 release (2010/11/23). Red rectangles are SNPs previously associated with COPD (Table 2). Blue rectangles are the most significant eQTL-SNPs for the four regulated genes found on 4q22 (Figure 1). The other illustrated SNPs were genotyped in our study and in LD (r^2^>0.5) with COPD SNPs.

**Figure 4:**
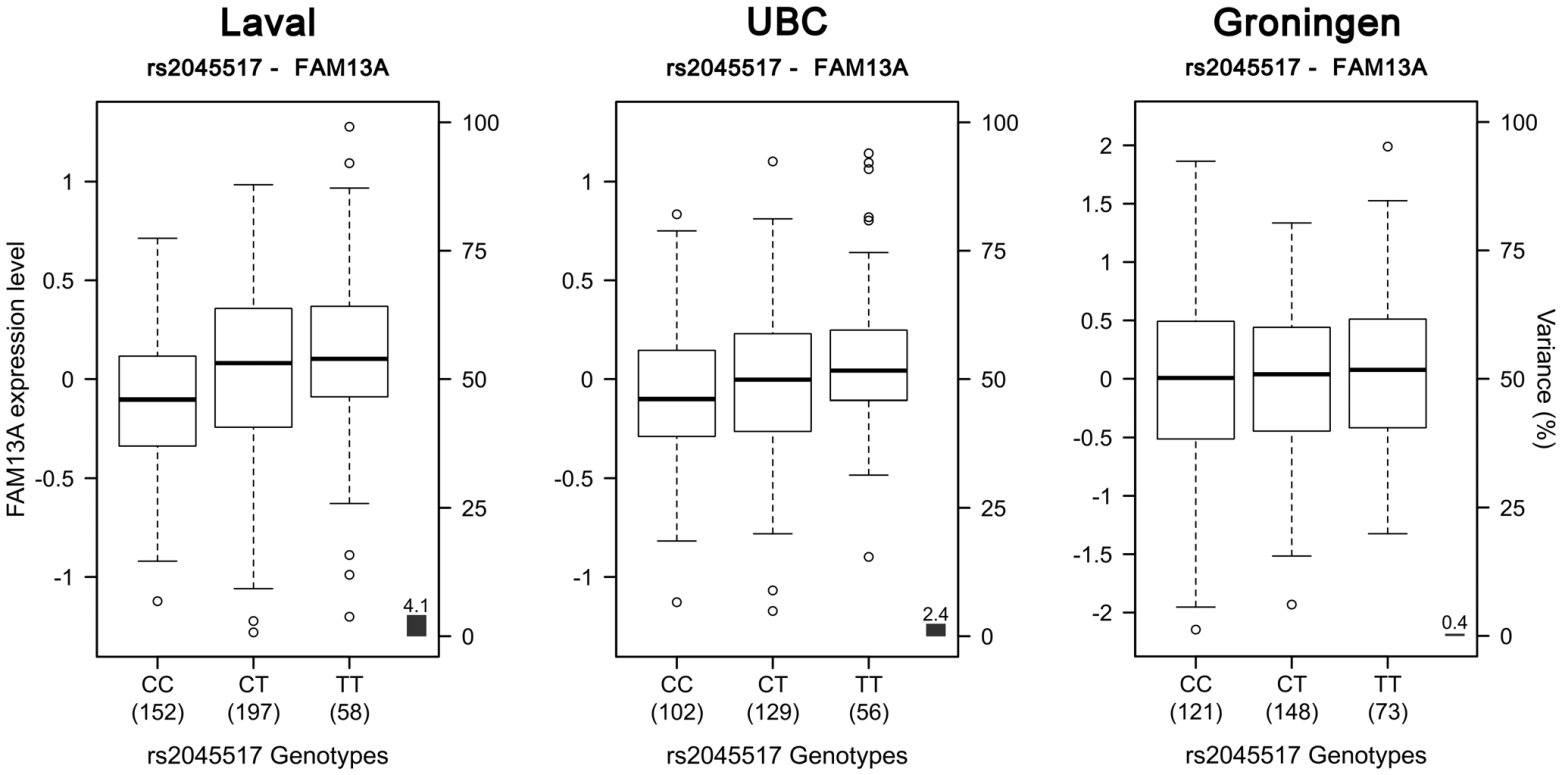
Boxplots of gene expression levels in the lung for *FAM13A* according to genotype groups for SNP rs2045517. The left y-axis shows the mRNA expression levels for *FAM13A*. The x-axis represents the three genotype groups for SNP rs2045517. The right y-axis shows the proportion of the gene expression variance explained by the SNP (black bar). Each panel represents a different cohort: Laval (n = 407), UBC (n = 287), Groningen (n = 342). The eQTL p values were 4.1×10^−5^, 0.009 and 0.218, respectively.

718 SNPs and 50 probesets covering 14 genes were located in the defined region on chromosome 4q22. 91 eQTLs were detected in the Laval set (Figure 1, Table S1). These 91 eQTLs consisted of 64 unique SNPs, 8 probesets and 4 genes (*PPM1K, GPRIN3*, *SNCA, MMRN1*). Significant linkage disequilibrium (LD) was observed among the 64 SNPs (Figure S1). 61 of the 91 eQTLs replicated in both replication cohorts (P<0.05). The strongest association detected in all cohorts was rs17013978 with *PPM1K* (Figure 1). The expression level of this gene decreased with the number of T alleles (Figure 2). In the three cohorts, this SNP explained 50.2 to 57.1% of the expression variance of *PPM1K* and the direction of the effect was the same in the three cohorts. None of the SNPs previously associated with COPD on 4q22 (Table 2) were genotyped in our eQTL dataset, but five of them were found in LD (r^2^>0.5) (Figure 3). These five SNPs did not significantly associated with the expression of genes at that locus, but a trend was observed with *FAM13A*. In fact, three of those five polymorphisms, in complete LD with each other (rs2045517, rs2869967, rs2869966) and in modest LD (r^2^ = 0.53–0.69) with COPD SNPs were nominally associated with the expression levels of *FAM13A* (p = 4.1×10^−5^). The *FAM13A*-rs2045517 eQTL was replicated in UBC, but not in Groningen (Figure 4).

### Lung eQTLs in the 4q31 Locus

**Figure 5:**
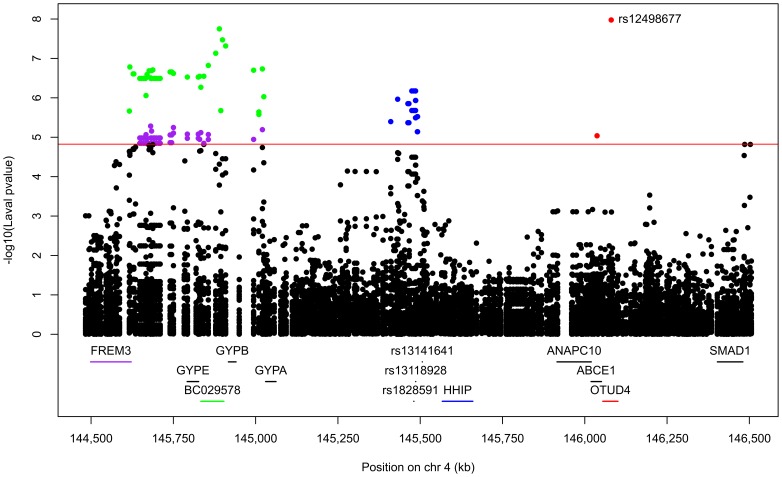
Lung eQTLs on 4q31 in the Laval dataset. Each dot represents an association test between one SNP and one probeset. Only dots above the red line are significant (p<1.5×10^−5^). Significant SNPs were regulating the expression levels of *BC029578* in green, *FREM3* in purple, *HHIP* in blue, and *OTUD4* in red. The SNP with the smaller p-value is indicated. SNPs previously associated with COPD are presented at the bottom.

**Figure 6:**
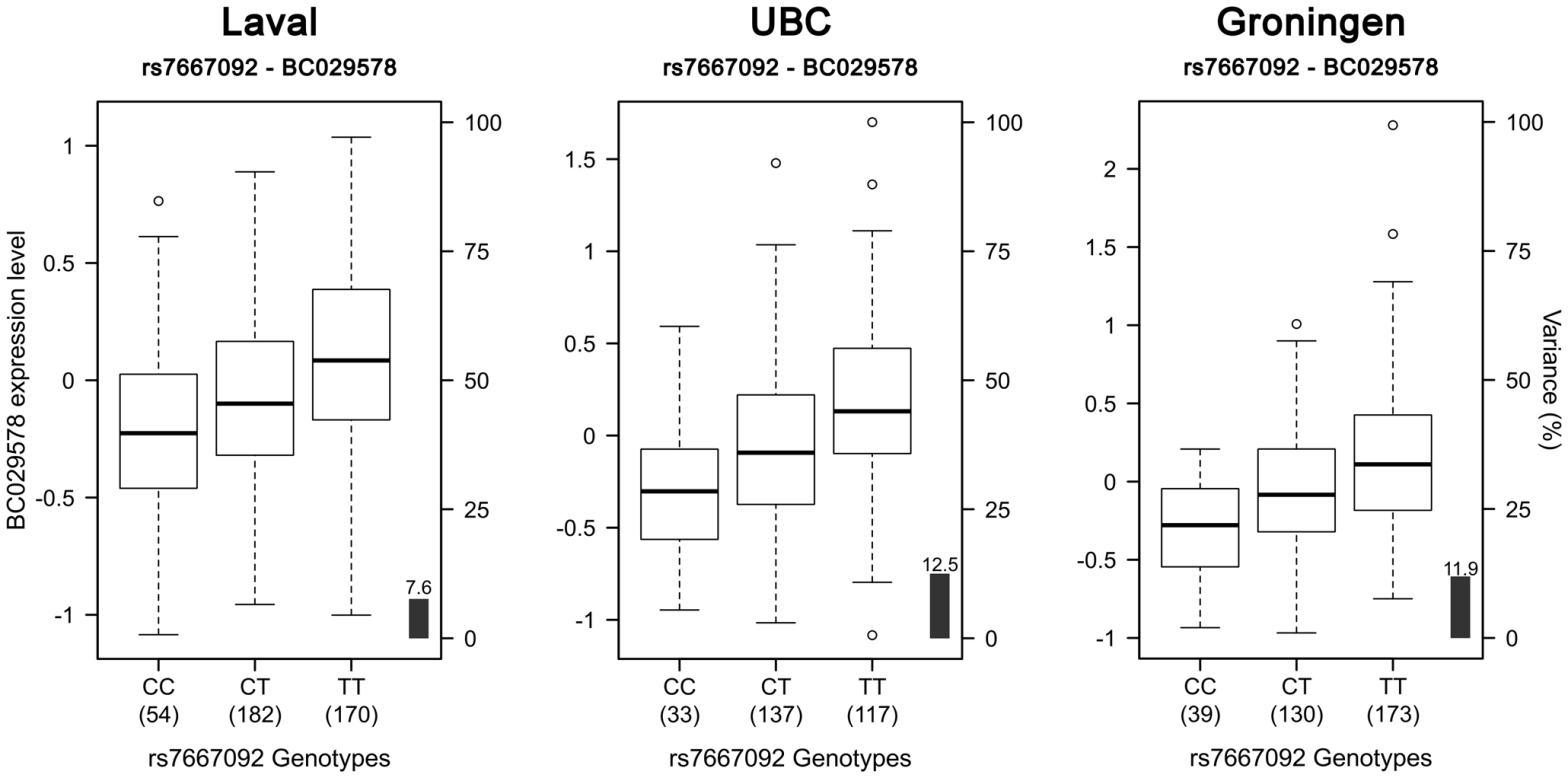
Boxplots of gene expression levels in the lung for *BC029578* according to genotype groups for SNP rs7667092. The left y-axis shows the mRNA expression levels for *BC029578*. The x-axis represents the three genotype groups for SNP rs7667092. The right y-axis shows the proportion of the gene expression variance explained by the SNP (black bar). Each panel represents a different cohort: Laval (n = 406), UBC (n = 287), Groningen (n = 342). The eQTL p-values were 1.8×10^−8^, 7.3×10^−10^ and 6.0×10^−11^, respectively.

**Figure 7:**
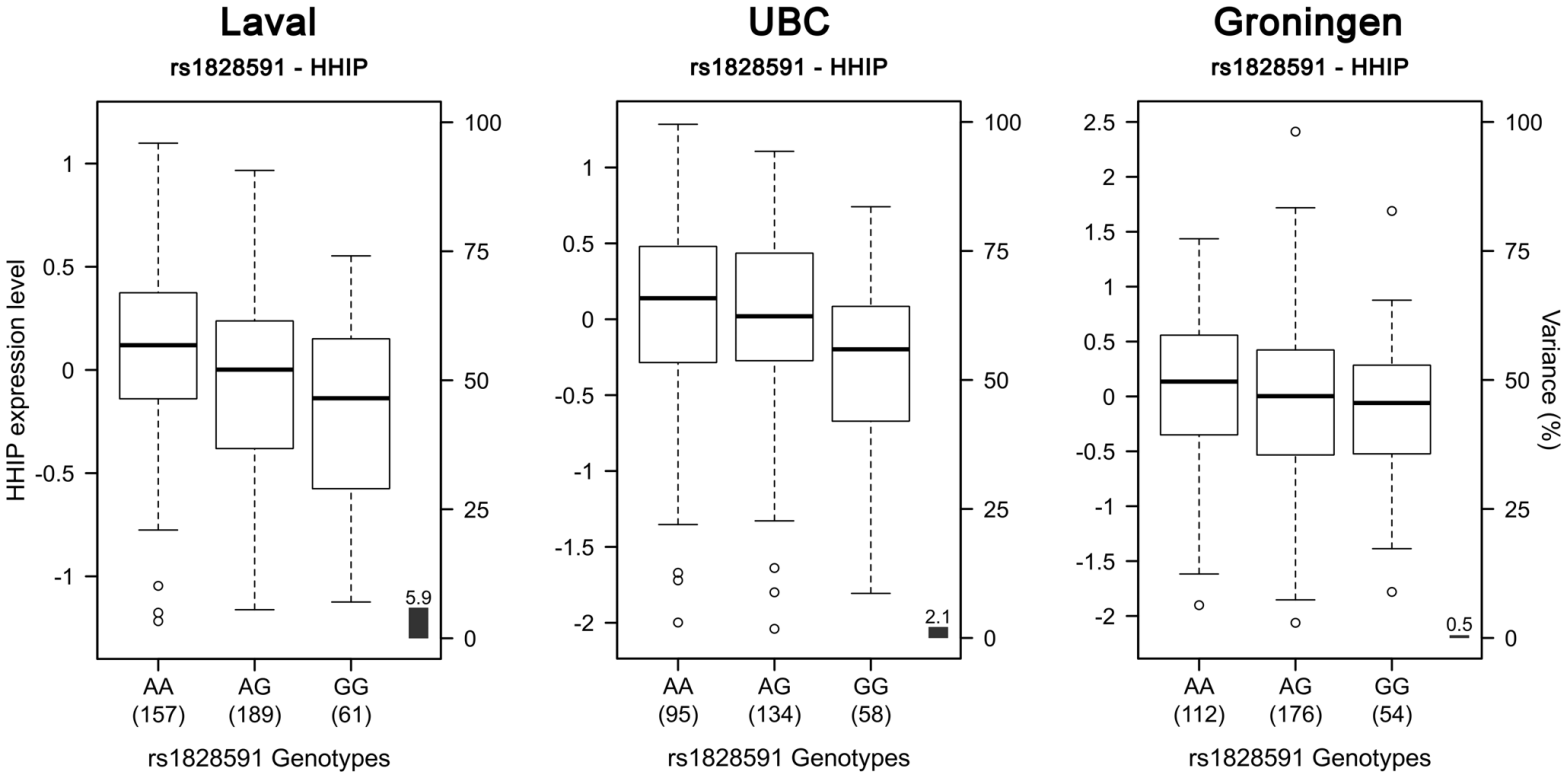
Boxplots of gene expression levels in the lung for *HHIP* according to genotype groups for SNP rs1828591. The left y-axis shows the mRNA expression levels for *HHIP*. The x-axis represents the three genotype groups for SNP rs1828591. The right y-axis shows the proportion of the gene expression variance explained by the SNP (black bar). Each panel represents a different cohort: Laval (n = 407), UBC (n = 287), Groningen (n = 342). The eQTL p-values were 6.7×10^−7^, 0.013 and 0.214, respectively.

412 SNPs and 34 probesets interrogating 9 unique genes were tested around previously COPD associated SNPs on chromosome 4q31. Significant eQTLs found in the Laval dataset are shown in Figure 5 and Table S2. 55 unique SNPs, 6 probesets and 4 genes (*FREM3*, *BC029578*, *HHIP*, *OTUD4*) were involved in the significant eQTLs. Only eQTLs associated with *BC029578* (35) and *OTUD4* (1) were replicated in the two replication sets. eQTL-SNPs on chromosome 4q31 are subdivided in two strong LD blocks (Figure S2). The strongest eQTL in Laval dataset, validated in both replication sets, was rs7667092 with *BC029578* (Figure 6). The expression levels of the gene increased with the number of T alleles in all cohorts. In the three cohorts, this SNP explained 7.6 to 12.5% of the gene expression variance of *BC029578*. However, this polymorphism was not in LD with SNPs previously associated with COPD (r^2^ = 0.016). Two SNPs (rs1828591, rs13118928) previously associated with COPD were found to affect the expression of *HHIP*. Rs1828591 was the most significant SNP associated with *HHIP* in the Laval dataset. This eQTL was replicated in UBC, but not in Groningen (Figure 7). The G allele was associated with lower expression of *HHIP* in the Laval and UBC datasets. The same pattern was observed in the Groningen set, but the association was not significant.

### Lung eQTLs in the 19q13 Locus

**Figure 8:**
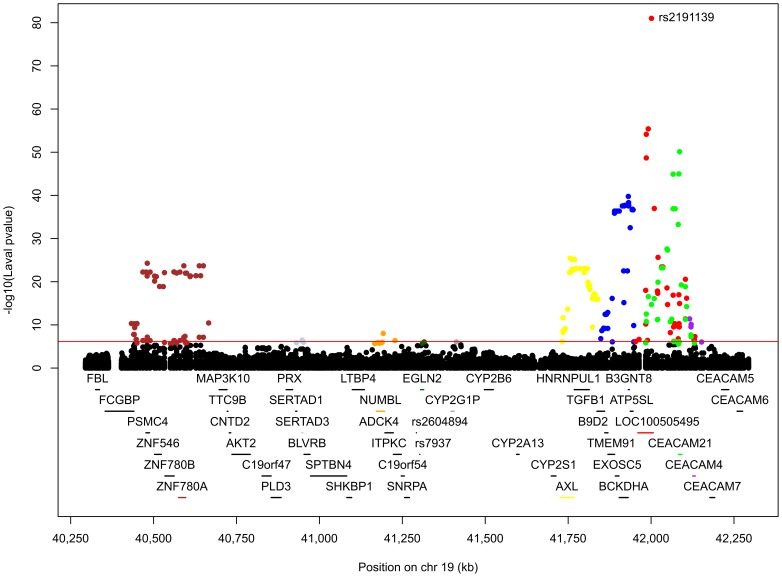
Lung eQTLs on 19q13 in the Laval dataset. Each dot represents an association test between one SNP and one probeset. Only dots above the red line are significant (p<7.1×10^−7^). Significant SNPs were regulating the expression levels of *ZNF780A* in brown, *SERTAD3* in light blue, *NUMBL* in orange, *EGLN2* in dark green, *CYP2G1P* in dark grey, *AXL* in yellow, *B3GNT8* in blue, *LOC100505495* in red, *CEACAM21* in green, and *CEACAM4* in purple. The SNP with the smaller p-value is indicated. SNPs previously associated with COPD are illustrated at the bottom.

**Figure 9:**
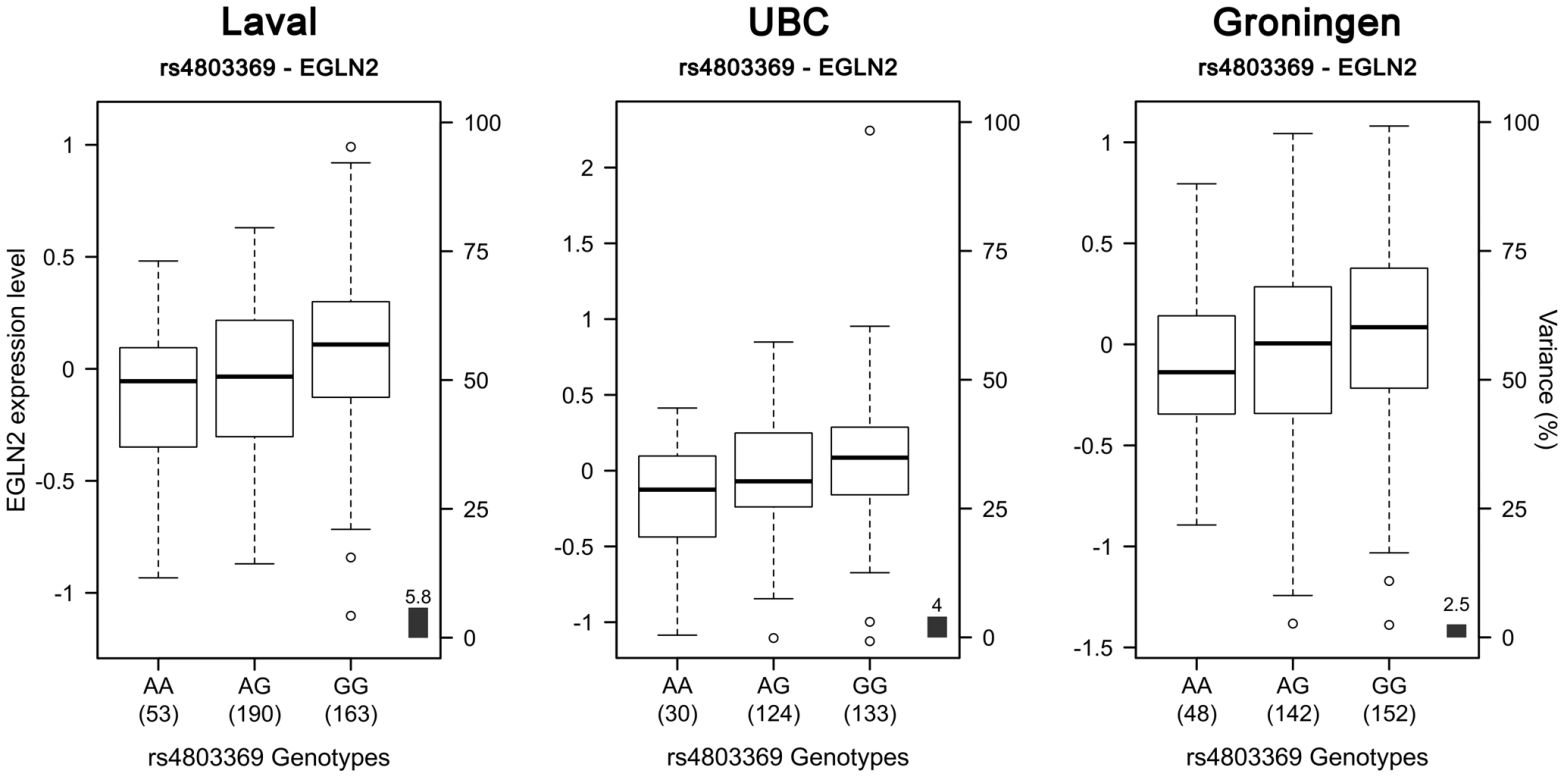
Boxplots of gene expression levels in the lung for *EGLN2* according to genotype groups for SNP rs4803369. The left y-axis shows the mRNA expression levels for *EGLN2*. The x-axis represents the three genotype groups for SNP rs4803369. The right y-axis shows the proportion of the gene expression variance explained by the SNP (black bar). Each panel represents a different cohort: Laval (n = 406), UBC (n = 287), Groningen (n = 342). The eQTL p values were 8.9×10^−7^, 0.001 and 0.003, respectively.

On 19q13, 739 SNPs and 95 probesets covering 45 different genes were tested. The expression levels of *RAB4B*, *MIA* and *CYP2A6* were not available in our datasets. 222 eQTLs were detected (Figure 8 and Table S3). 174 SNPs were regulating 11 probesets located on 10 genes (*ZNF780A, SERTAD3, NUMBL, EGLN2, CYP2G1P, AXL*, *B3GNT8*, *LOC100505495*, *CEACAM21*, *CEACAM4*). 210 eQTLs were validated in both replication cohorts. SNPs associated with gene expression were distributed across four LD blocks (Figure S3). 26 SNPs were associated with the expression levels of *CEACAM21* and *LOC100505495*, and 3 others SNPs were associated with *CEACAM21* and *CEACAM4*. The eQTLs on 19q13 were mainly located in two discrete foci one distal and one proximal to the COPD susceptibility locus *RAB4B/EGLN2/MIA/CYP2A6* (Figure 8). These eQTL-SNPs were not in LD with the COPD SNPs rs7937 and rs2604894. The latter two SNPs were in strong LD (r^2^ = 0.82) and rs7937 was genotyped in our lung eQTL dataset. Rs7937 was not associated with expression of genes located in this predefined 19q13 locus. The gene most significantly regulated by rs7937 was *NUMBL* (p = 0.0187). Three SNPs were regulating the expression of *EGLN2*, a gene previously associated with COPD. The most significant association with *EGLN2* was with rs4803369 (p = 8.9×10^−7^). Rs4803369 is located at 13,274 bp away from rs7937 and is in modest LD (r^2^ = 0.33) with the latter. The eQTL results for rs4803369-*EGLN2* from the three cohorts are illustrated in Figure 9. This eQTL was significant and had the same direction of effect in all 3 cohorts.

## Discussion

The goal of this study was to identify causal variants and genes within susceptibility loci associated with COPD. GWAS have indicated four loci associated with this disease as defined by lung function measures [9]–[11]. However, GWAS could not fully revealed the genetic mechanisms mediating the risk within these loci. In this study, we used genotypes and expression values of a large number of lung samples derived from three independent populations to identify eQTLs. Our analyses were centered on three loci previously associated with COPD: 4q22 (*FAM13A*), 4q31 (*HHIP*) and 19q13 (*RAB4B*, *EGLN2*, *MIA*, *CYP2A6*). We identified genetic variants influencing gene expression at each locus and replicated findings in two independent cohorts.

The first study to identify an association between the 4q22 locus and COPD was published in 2010 [19]. Three other studies confirmed an association between this locus and COPD/lung function [10], [11], [20]. In this study, we found 91 eQTLs on 4q22 in the discovery cohort and 61 of them were replicated in both replication sets. The majority of the SNPs were located in introns (n = 30) and intergenic regions (n = 27). Other SNPs were located in the 3′ UTR (n = 4) and upstream of the gene (n = 2). Only one missense SNP was found to regulate the expression of *MMRN1*. Lung eQTLs on 4q22 were found and validated with four genes: *PPM1K*, *SNCA*, *PPM1* and *GPRIN3* genes. A SNP located in SNCA (rs2035268) has been associated with accelerated FEV1/FVC decline [21]. Three SNPs in our dataset were in perfect LD with the rs2035268: rs3889917, rs7684637 and rs3775461. However, none of those SNPs were significantly associated with the expression level of a gene. The best r^2^ between one of our significant eQTL-SNP and rs2035268 was 0.047. No SNP previously associated with COPD within and near *FAM13A* [10], [11], [19] were available in our dataset. However, some polymorphisms were in LD with the disease associated SNPs (r^2^>0.5). The latter were nominally associated with the expression of *FAM13A* and validated in one replication set. Accordingly, our results provide some support that *FAM13A* is the COPD causal gene on 4q22.

The 4q31 locus was first associated with COPD and lung function in two studies in 2009 [9], [22], and then replicated in four other GWAS [10], [11], [19], [23]. In our discovery set, 55 SNPs, 6 probesets covering 4 genes were significant. Interestingly, some of the eQTL-SNPs have been previously associated with COPD (rs1828591 [9], [24], rs13118928 [9], [24]) and lung function (rs1828591 [22], rs7655625 [22], rs1980057 [19], [22]). SNPs were mainly located in intergenic regions (n = 49). Others were in introns (n = 4), coding-synonymous region (n = 1) and 3′UTR region (n = 1). The only eQTLs replicating in the two replication sets are those associated with the *BC029578* transcript and another associated with *OTUD4*. This transcript is located between the *GYPE* and *GYPB* genes. SNPs regulating *BC029578* are distributed across a 400 kb region. Further studies are needed to understand the function of *BC029578*. eQTLs were also associated with *FREM3* and *HHIP*, a member of the hedgehog-interacting protein family. *HHIP* has been associated with COPD in three GWAS [9]–[11]. Significant eQTLs in this gene only replicated in UBC, but the same direction of effect was also observed in the Goningen set. These results supported that HHIP is the most likely causal gene in the region.

There are many genes present in the 19q13 locus. This locus was recently associated with COPD and so far no replication study has been published [11]. 222 eQTLs were detected in our original set and 210 of them were validated in the replication sets. Ten genes were regulated by SNPs in the Laval dataset, which were all validated in replication sets. Some SNPs have been previously associated with COPD (rs2302188 [25], rs4803481 [25], rs1800469 [26], [27]) and lung function (rs2241718 [26], rs6957 [26]). Interestingly, *CEACAM21* was associated with COPD susceptibility in a sputum eQTLs study on COPD patients [25]. This gene encodes carcinoembryonic antigen, who has been found to be overexpressed in heavy smokers [28], [29]. To the best of our knowledge, no studies have to date supported the contribution of *AXL*, *NUMBL*, *SERTAD3*, *B3GNT8*, *CEACAM4*, CYP2G1P, *LOC100505495* or *ZNF780A* to the development of COPD or related phenotypes. Rs7937, a SNP located in *RAB4B*, *EGLN2* and *MIA-RAB4B* and identified in previous GWAS, was genotyped in our datasets. However, no association was detected between this SNP and the expression level of any gene. The strongest association with a suspected COPD gene is *EGLN2*-rs4803369. This gene is known to be involved in regulating hypoxia tolerance and apoptosis in cardiac and skeletal muscle. These results support that *EGLN2* is a potential causal COPD gene on 19q13.

eQTLs obtained in this study are derived from non-tumor lung parenchymal samples. As all organs, the lung is multicellular. The cellular heterogeneity constitutes an inherent limitation of our study and will inevitably reduce the power to detect eQTLs. It is known that many eQTLs will be missed by studying heterogeneous tissues [30]. Although many eQTLs are shared across tissues [31], [32], a relatively large portion of eQTLs are cell type- and tissue-specific [33], [34]. eQTL mapping results are also known to be affected by environmental cues as well as the development stage and differentiation states of cells [35], [36]. Due to the spatiotemporal characteristics of eQTLs [30]–[38], the lung eQTL results in this study will need to be verified in others disease-relevant tissues and cell types as well as in tissues from diseased and healthy individuals.

In conclusion, we used a large collection of human lung specimens from patients with and without COPD to identify SNPs that regulated gene expression in three COPD susceptibility loci derived from GWAS. Strong lung eQTLs were detected in the three COPD loci. However, the eQTL-SNPs were not necessarily the SNPs associated with COPD. On 4q22, SNPs associated with COPD near the *FAM13A* gene were indirectly (in LD) associated with the mRNA expression levels of *FAM13A*. On 4q31, the suspected candidate in this region, *HHIP*, was found to be regulated by SNPs previously associated with COPD. On 19q13, SNPs associated with COPD were consistently associated with the expression level of *EGLN2*. Further functional studies will be needed to verify the contribution of susceptibility genes in COPD. Strong lung eQTL SNPs will also need to be tested for association with COPD in case-control studies and in additional eQTL mapping studies in other disease-relevant tissues and cell types. This study is an important step forward to better understanding the underlying biology of the COPD susceptibility loci. It also shows the potential of eQTLs in a relevant tissue to leverage the results of previous GWAS and extend their functional meaning to gene expression.

## Supporting Information

Figure S1 **Linkage disequilibrium plot of significant SNPs on the 4q22 locus in the 1000 Genome Project.** The white horizontal bar on the upper part of the figure illustrates the location of SNPs on a physical scale. LD values (r^2^) are indicated in each box. The color of the squares illustrate the strength of pairwise r^2^ values on a black and white scale where black indicates perfect LD (r^2^ = 1) and white indicates perfect equilibrium (r^2^ = 0). Red rectangles are SNPs previously associated with COPD (Table 2). The genotypes are from the 1000 Genome Project interim phase1 release (2010/11/23).
(TIFF)

Figure S2 **Linkage disequilibrium plot of significant SNPs on the 4q31 locus in the 1000 Genome Project.** The white horizontal bar on the upper part of the figure illustrates the location of SNPs on a physical scale. LD values (r^2^) are indicated in each box. The color of the squares illustrate the strength of pairwise r^2^ values on a black and white scale where black indicates perfect LD (r^2^ = 1) and white indicates perfect equilibrium (r^2^ = 0). Red rectangles are SNPs previously associated with COPD (Table 2). The genotypes are from the 1000 Genome Project interim phase1 release (2010/11/23).
(TIFF)

Figure S3 **Linkage disequilibrium plot of significant SNPs on the 19q13 locus in the 1000 Genome Project.** The white horizontal bar on the upper part of the figure illustrates the location of SNPs on a physical scale. LD values (r^2^) are indicated in each box. The color of the squares illustrate the strength of pairwise r^2^ values on a black and white scale where black indicates perfect LD (r^2^ = 1) and white indicates perfect equilibrium (r^2^ = 0). Red rectangles are SNPs previously associated with COPD (Table 2). The genotypes are from the 1000 Genome Project interim phase1 release (2010/11/23).
(TIFF)

Table S1 **Significant eQTLs at the 4q22 locus in the Laval dataset and replication in UBC and Groningen datasets.**
(DOCX)

Table S2 **Significant eQTLs at the 4q31 locus in the Laval dataset and replication in UBC and Groningen datasets.**
(DOCX)

Table S3 **Significant eQTLs at the 19q13 locus in the Laval dataset and replication in UBC and Groningen datasets.**
(DOCX)
