# Supplementary figures and images for: Refining Susceptibility Loci of Chronic Obstructive Pulmonary Disease with Lung eqtls
Source: PLoS One. 2013 Jul 30;8(7):e70220. doi: 10.1371/journal.pone.0070220 (PMC3728203; doi:10.1371/journal.pone.0070220)

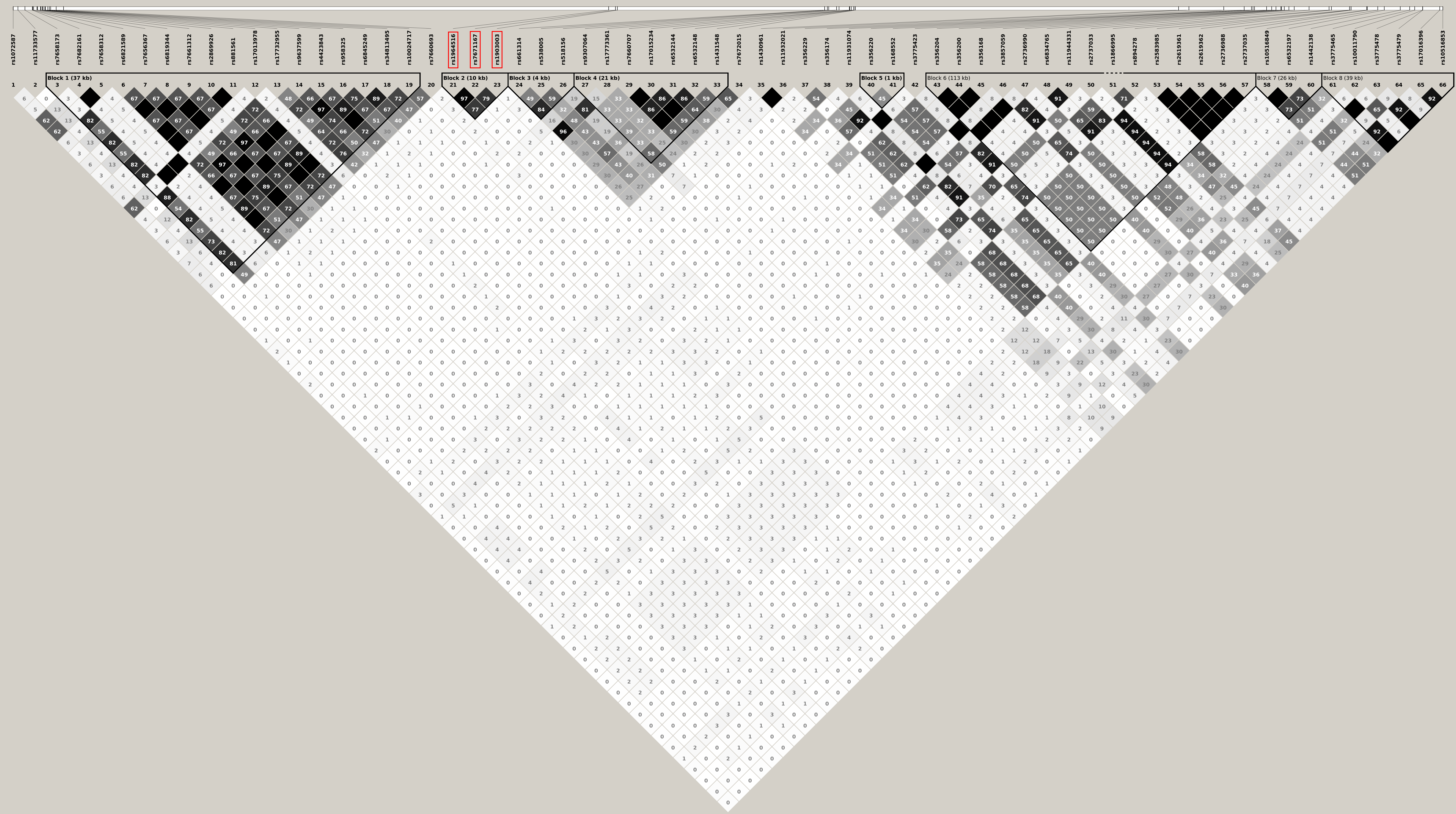

Supplement: Figure S1 — Linkage disequilibrium plot of significant SNPs on the 4q22 locus in the 1000 Genome Project. The white horizontal bar on the upper part of the figure illustrates the location of SNPs on a physical scale. LD values (r2) are indicated in each box. The color of the squares illustrate the strength of pairwise r2 values on a black and white scale where black indicates perfect LD (r2 = 1) and white indicates perfect equilibrium (r2 = 0). Red rectangles are SNPs previously associated with COPD (Table 2). The genotypes are from the 1000 Genome Project interim phase1 release (2010/11/23). (TIFF) [file pone.0070220.s001.tiff]

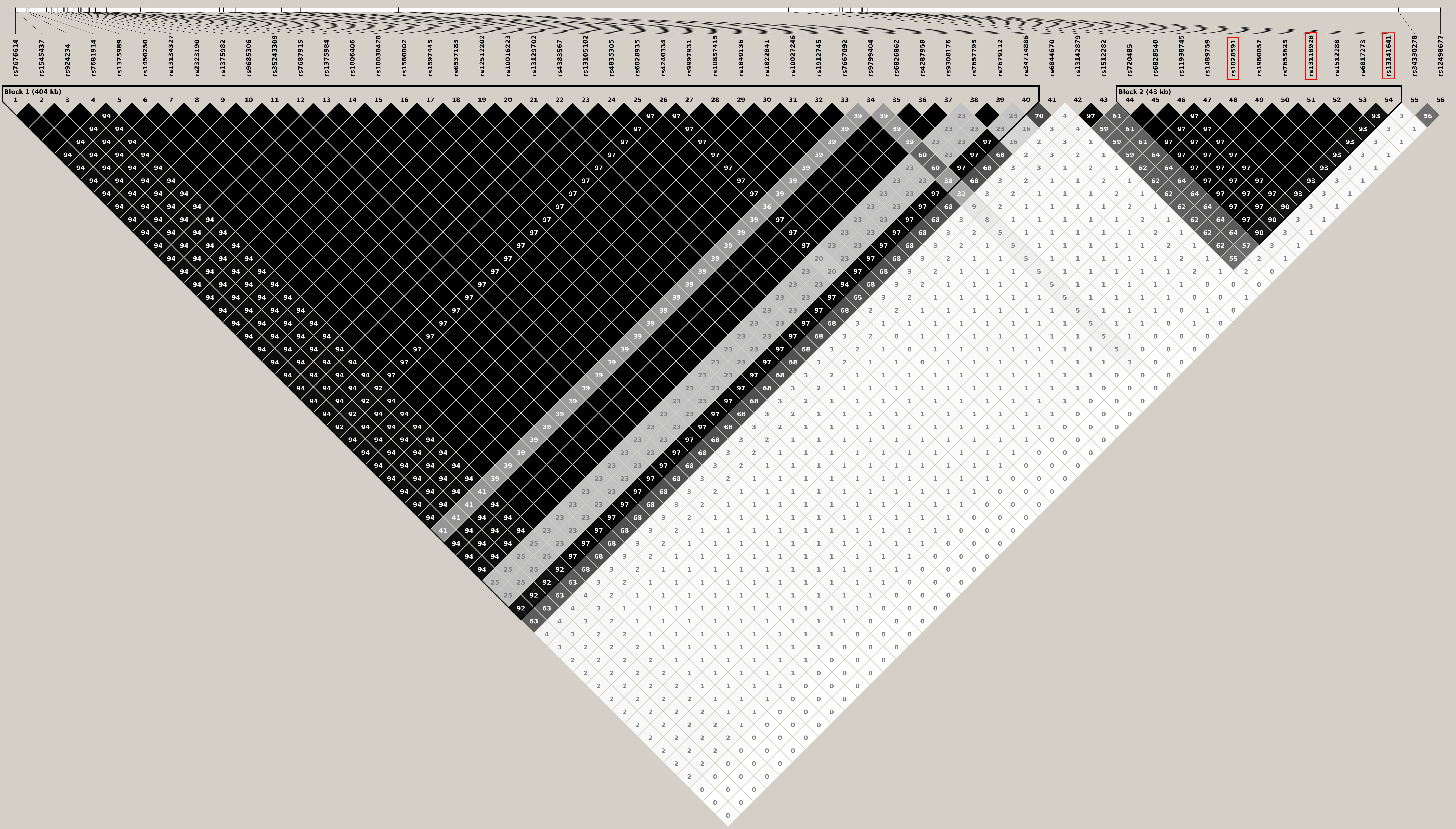

Supplement: Figure S2 — Linkage disequilibrium plot of significant SNPs on the 4q31 locus in the 1000 Genome Project. The white horizontal bar on the upper part of the figure illustrates the location of SNPs on a physical scale. LD values (r2) are indicated in each box. The color of the squares illustrate the strength of pairwise r2 values on a black and white scale where black indicates perfect LD (r2 = 1) and white indicates perfect equilibrium (r2 = 0). Red rectangles are SNPs previously associated with COPD (Table 2). The genotypes are from the 1000 Genome Project interim phase1 release (2010/11/23). (TIFF) [file pone.0070220.s002.tiff]
