# Supplementary material for: Refining Susceptibility Loci of Chronic Obstructive Pulmonary Disease with Lung eqtls
Source: PLoS One. 2013 Jul 30;8(7):e70220. doi: 10.1371/journal.pone.0070220 (PMC3728203; doi:10.1371/journal.pone.0070220)
Supplement: Table S1 — Significant eQTLs at the 4q22 locus in the Laval dataset and replication in UBC and Groningen datasets. (DOCX) [file pone.0070220.s004.docx]

**Table S1: Significant eQTLs at the 4q22 locus in the Laval dataset and replication in UBC and Groningen datasets.**

| **SNP** | **SNP Position** | **SNP Function** | **Regulated Gene** | **P Laval** | **eQTL Direction  Laval*** | **P UBC** | **eQTL Direction  UBC*** | **P Groningen** | **eQTL Direction  Groningen*** |
| --- | --- | --- | --- | --- | --- | --- | --- | --- | --- |
| rs17013978 | 89203197 | intron | PPM1K | 5.636E-061 | TT-CT | 2.788E-054 | TT-CT-CC | 3.765E-058 | TT-CT |
| rs7658312 | 89191741 | intron | PPM1K | 2.876E-060 | CC-TC | 2.788E-054 | CC-TC-TT | 3.765E-058 | CC-TC |
| rs7682161 | 89191346 | intron | PPM1K | 2.876E-060 | TT-CT | 2.788E-054 | TT-CT-CC | 3.765E-058 | TT-CT |
| rs6532144 | 90164136 | unknown | GPRIN3 | 3.167E-014 | TT-CT-CC | 0.000001629 | TT-CT-CC | 0.003492 | TT-CT-CC |
| rs7660707 | 90161863 | unknown | GPRIN3 | 2.319E-013 | TT-CT-CC | 1.734E-009 | TT-CT-CC | 0.0006004 | TT-CT-CC |
| rs17015234 | 90162268 | unknown | GPRIN3 | 2.656E-013 | CC-AC-AA | 1.734E-009 | CC-AC-AA | 0.0006968 | CC-AC-AA |
| rs6532148 | 90166921 | untranslated-3 | GPRIN3 | 6.589E-013 | AA-GA-GG | 1.74E-009 | AA-GA-GG | 0.0006968 | AA-GA-GG |
| rs1431548 | 90168572 | untranslated-3 | GPRIN3 | 1.078E-010 | GG-AG-AA | 0.0008351 | GG-AG-AA | 0.03055 | GG-AG-AA |
| rs538005 | 90135739 | unknown | GPRIN3 | 1.983E-010 | CC-TC-TT | 0.001258 | CC-TC-TT | 0.1749 | CC-TC-TT |
| rs2736988 | 90776345 | unknown | SNCA | 2.094E-010 | GG-AG-AA | 0.006627 | GG-AG-AA | 0.05687 | GG-AG-AA |
| rs2737035 | 90777213 | unknown | SNCA | 2.094E-010 | GG-AG-AA | 0.006627 | GG-AG-AA | 0.05687 | GG-AG-AA |
| rs11733577 | 89174193 | unknown | PPM1K | 2.459E-010 | TT-CT-CC | 3.394E-009 | TT-CT-CC | 9.054E-013 | TT-CT-CC |
| rs168552 | 90643144 | unknown | SNCA | 2.822E-010 | TT-CT-CC | 0.01156 | TT-CT-CC | 0.05368 | TT-CT-CC |
| rs894278 | 90734535 | intron | MMRN1 | 3.026E-010 | TT-GT | 0.000007273 | TT-GT | NA | NA |
| rs2619361 | 90757735 | intron | SNCA | 3.389E-010 | CC-AC-AA | 0.004987 | CC-AC-AA | 0.02271 | CC-AC-AA |
| rs2619362 | 90757845 | intron | SNCA | 3.389E-010 | CC-TC-TT | 0.004987 | CC-TC-TT | 0.02271 | CC-TC-TT |
| rs2583985 | 90755939 | intron | SNCA | 3.389E-010 | AA-GA-GG | 0.006661 | AA-GA-GG | 0.02271 | AA-GA-GG |
| rs356174 | 90630901 | unknown | SNCA | 4.897E-010 | AA-CA-CC | 0.02947 | AA-CC-CA | 0.1582 | AA-CA-CC |
| rs1866995 | 90731709 | intron | MMRN1 | 6.569E-010 | AA-GA-GG | 0.000001031 | AA-GA-GG | 0.0001333 | AA-GA |
| rs894278 | 90734535 | intron | MMRN1 | 1.165E-009 | TT-GT | 0.000005342 | TT-GT | NA | NA |
| rs2737033 | 90707947 | intron | SNCA | 1.277E-009 | AA-GA-GG | 0.006268 | AA-GA-GG | 0.05424 | AA-GA-GG |
| rs9307064 | 90146649 | unknown | GPRIN3 | 1.782E-009 | GG-GA-AA | 0.01899 | GA-GG-AA | 0.197 | GG-GA-AA |
| rs9637599 | 89206230 | near-gene-5 | PPM1K | 2.534E-009 | AA-CA-CC | 1.825E-009 | AA-CA-CC | 2.191E-008 | AA-CA-CC |
| rs11944331 | 90690329 | intron | MMRN1 | 2.611E-009 | CC-TC-TT | 0.0000252 | CC-TT-TC | 0.009669 | TT-CC-TC |
| rs3775423 | 90657491 | intron | MMRN1 | 2.611E-009 | CC-TC-TT | 0.0000252 | CC-TT-TC | 0.01135 | TT-CC-TC |
| rs3857059 | 90675238 | intron | MMRN1 | 2.611E-009 | AA-GA-GG | 0.0000252 | AA-GG-GA | 0.01135 | GG-AA-GA |
| rs11931074 | 90639515 | unknown | MMRN1 | 2.611E-009 | GG-TG-TT | 0.0002428 | GG-TT-TG | 0.01135 | TT-GG-TG |
| rs1072587 | 89168575 | unknown | PPM1K | 3.154E-009 | CC-TC | 5.78E-013 | CC-TC-TT | 1.054E-016 | CC-TT-TC |
| rs6834765 | 90683990 | intron | MMRN1 | 4.732E-009 | TT-CT-CC | 0.00002089 | TT-CC-CT | 0.003565 | CC-TT-CT |
| rs11944331 | 90690329 | intron | MMRN1 | 0.000000005 | CC-TC-TT | 0.00001595 | CC-TT-TC | 0.0005033 | CC-TT-TC |
| rs3775423 | 90657491 | intron | MMRN1 | 0.000000005 | CC-TC-TT | 0.00001595 | CC-TT-TC | 0.0006508 | CC-TT-TC |
| rs3857059 | 90675238 | intron | MMRN1 | 0.000000005 | AA-GA-GG | 0.00001595 | AA-GG-GA | 0.0006508 | AA-GG-GA |
| rs11931074 | 90639515 | unknown | MMRN1 | 0.000000005 | GG-TG-TT | 0.0001435 | GG-TT-TG | 0.0006508 | GG-TT-TG |
| rs1866995 | 90731709 | intron | MMRN1 | 5.864E-009 | AA-GA-GG | 0.000002536 | AA-GA-GG | 5.605E-007 | AA-GA |
| rs958325 | 89209681 | unknown | PPM1K | 9.535E-009 | CC-TC-TT | 4.469E-010 | CC-TC-TT | 7.158E-009 | CC-TC-TT |
| rs7661312 | 89201007 | intron | PPM1K | 1.121E-008 | TT-GT-GG | 1.816E-009 | TT-GT-GG | 0.000000031 | TT-GT-GG |
| rs7656367 | 89196821 | intron | PPM1K | 1.172E-008 | AA-GA-GG | 1.816E-009 | AA-GA-GG | 0.000000031 | AA-GA-GG |
| rs168552 | 90643144 | unknown | SNCA | 1.219E-008 | TT-CT-CC | 0.01146 | TT-CT-CC | 0.1713 | TT-CT-CC |
| rs356174 | 90630901 | unknown | SNCA | 1.569E-008 | AA-CA-CC | 0.03077 | AA-CA-CC | 0.3699 | AA-CA-CC |
| rs168552 | 90643144 | unknown | SNCA | 1.886E-008 | TT-CT-CC | 0.03586 | TT-CT-CC | 0.2425 | TT-CT-CC |
| rs7658173 | 89182518 | untranslated-3 | PPM1K | 2.931E-008 | TT-TC-CC | 3.837E-009 | TT-TC-CC | 0.0000443 | TT-TC-CC |
| rs356174 | 90630901 | unknown | SNCA | 3.042E-008 | AA-CA-CC | 0.04897 | AA-CA-CC | 0.5177 | AA-CA-CC |
| rs10516849 | 90789539 | unknown | MMRN1 | 3.159E-008 | AA-GA-GG | 0.000007747 | AA-GA-GG | 0.000022 | AA-GG-GA |
| rs6532197 | 90797301 | unknown | MMRN1 | 3.159E-008 | AA-GA-GG | 0.000007747 | AA-GA-GG | 0.000022 | AA-GG-GA |
| rs356220 | 90641340 | unknown | SNCA | 3.383E-008 | CC-TC-TT | 0.1447 | CC-TC-TT | 0.05151 | CC-TC-TT |
| rs2869926 | 89201376 | intron | PPM1K | 3.744E-008 | TT-TC-CC | 7.721E-008 | TT-TC-CC | 0.00009052 | TT-TC-CC |
| rs6821589 | 89192792 | intron | PPM1K | 3.744E-008 | GG-GA-AA | 7.721E-008 | GG-GA-AA | 0.00009052 | GG-GA-AA |
| rs881561 | 89203042 | intron | PPM1K | 3.744E-008 | AA-AG-GG | 7.721E-008 | AA-AG-GG | 0.00009052 | AA-AG-GG |
| rs6819344 | 89197519 | intron | PPM1K | 4.28E-008 | AA-AC-CC | 7.721E-008 | AA-AC-CC | 0.00009052 | AA-AC-CC |
| rs6834765 | 90683990 | intron | MMRN1 | 4.331E-008 | TT-CT-CC | 0.00001477 | TT-CC-CT | 0.0000533 | TT-CC-CT |
| rs4423843 | 89206756 | near-gene-5 | PPM1K | 0.000000046 | GG-GA-AA | 0.000000084 | GG-GA-AA | 0.0001531 | GG-GA-AA |
| rs10516849 | 90789539 | unknown | MMRN1 | 6.817E-008 | AA-GA-GG | 0.00001662 | AA-GA-GG | 0.0008057 | GG-AA-GA |
| rs6532197 | 90797301 | unknown | MMRN1 | 6.817E-008 | AA-GA-GG | 0.00001662 | AA-GA-GG | 0.0008057 | GG-AA-GA |
| rs356220 | 90641340 | unknown | SNCA | 1.595E-007 | CC-TC-TT | 0.1094 | CC-TC-TT | 0.2813 | CC-TT-TC |
| rs10024717 | 89219645 | unknown | PPM1K | 2.146E-007 | AA-AG-GG | 5.844E-007 | AA-AG-GG | 0.00009618 | AA-AG-GG |
| rs34813495 | 89213077 | unknown | PPM1K | 2.159E-007 | GG-GA-AA | 0.000002308 | GG-GA-AA | 0.0002057 | GG-GA-AA |
| rs3775465 | 90827466 | intron | MMRN1 | 2.618E-007 | AA-CA-CC | 6.313E-007 | AA-CA-CC | 0.002258 | CC-AA-CA |
| rs7660693 | 89228383 | unknown | PPM1K | 2.992E-007 | CC-CT-TT | 0.000007022 | CC-TC-TT | 0.02183 | CC-TC-TT |
| rs28661946 | 89214757 | unknown | PPM1K | 3.093E-007 | AA-AT-TT | 3.35E-009 | AA-AT-TT | 0.000000353 | AA-AT-TT |
| rs1442138 | 90816294 | missense | MMRN1 | 3.912E-007 | TT-CT-CC | 0.000009703 | TT-CT-CC | 0.000002381 | TT-CT |
| rs10011790 | 90833421 | intron | MMRN1 | 4.141E-007 | GG-GA-AA | 0.04635 | GG-GA-AA | 0.001688 | GG-GA-AA |
| rs518156 | 90137336 | unknown | GPRIN3 | 5.796E-007 | CC-AC-AA | 0.08178 | CC-AC-AA | 0.8506 | AC-CC-AA |
| rs1442138 | 90816294 | missense | MMRN1 | 5.875E-007 | TT-CT-CC | 0.000009961 | TT-CT-CC | 0.00008171 | TT-CT |
| rs3775479 | 90842971 | intron | MMRN1 | 5.935E-007 | CC-CA-AA | 0.0283 | CC-CA-AA | 0.002504 | CC-AC-AA |
| rs2737033 | 90707947 | intron | SNCA | 7.563E-007 | AA-GA-GG | 0.04443 | AA-GA-GG | 0.297 | AA-GA-GG |
| rs3775478 | 90842840 | intron | MMRN1 | 7.743E-007 | AA-GA-GG | 0.000007712 | AA-GA-GG | 0.002843 | GG-AA-GA |
| rs661314 | 90132470 | unknown | GPRIN3 | 0.000001685 | CC-TC-TT | 0.003872 | CC-TC-TT | 0.8956 | TC-CC-TT |
| rs17732955 | 89204093 | intron | PPM1K | 0.000001788 | TT-TC-CC | 3.964E-008 | TT-TC-CC | 0.0001193 | TT-TC-CC |
| rs17773361 | 90149589 | unknown | GPRIN3 | 0.000002224 | AA-CA-CC | 0.002699 | AA-CA-CC | 0.5334 | AA-CA-CC |
| rs2619361 | 90757735 | intron | SNCA | 0.000002246 | CC-AC-AA | 0.0549 | CC-AC-AA | 0.2584 | CC-AC-AA |
| rs2619362 | 90757845 | intron | SNCA | 0.000002246 | CC-TC-TT | 0.0549 | CC-TC-TT | 0.2584 | CC-TC-TT |
| rs2583985 | 90755939 | intron | SNCA | 0.000002246 | AA-GA-GG | 0.05725 | AA-GA-GG | 0.2584 | AA-GA-GG |
| rs356220 | 90641340 | unknown | SNCA | 0.00000237 | CC-TT-TC | 0.2275 | CC-TC-TT | 0.4877 | CC-TC-TT |
| rs7661312 | 89201007 | intron | PPM1K | 0.000002566 | GG-GT-TT | 0.000008296 | GG-GT-TT | 0.0002818 | GG-GT-TT |
| rs10516853 | 90866819 | intron | MMRN1 | 0.00000258 | CC-TC-TT | 0.000005111 | CC-TC-TT | 0.01115 | TT-CC-TC |
| rs7672015 | 90168825 | untranslated-3 | GPRIN3 | 0.000002609 | TT-CT-CC | 0.0128 | TT-CT-CC | 0.04691 | TT-CT-CC |
| rs356229 | 90606597 | unknown | SNCA | 0.000003022 | AA-GA-GG | 0.4095 | AA-GG-GA | 0.2243 | AA-GG-GA |
| rs11932021 | 90565218 | unknown | MMRN1 | 0.000003127 | CC-TC-TT | 0.0004345 | CC-TT-TC | 0.005562 | TT-CC-TC |
| rs356229 | 90606597 | unknown | SNCA | 0.00000313 | AA-GG-GA | 0.3402 | AA-GA-GG | 0.621 | AA-GG-GA |
| rs17016396 | 90863170 | intron | MMRN1 | 0.000003442 | TT-CT-CC | 0.00001461 | TT-CT-CC | 0.01536 | CC-TT-CT |
| rs11932021 | 90565218 | unknown | MMRN1 | 0.000003797 | CC-TC-TT | 0.001779 | CC-TT-TC | 0.0001479 | TT-CC-TC |
| rs2736988 | 90776345 | unknown | SNCA | 0.000004166 | GG-AG-AA | 0.06272 | GG-AG-AA | 0.2337 | GG-AG-AA |
| rs2737035 | 90777213 | unknown | SNCA | 0.000004166 | GG-AG-AA | 0.06272 | GG-AG-AA | 0.2337 | GG-AG-AA |
| rs6845249 | 89211522 | unknown | PPM1K | 0.000004261 | TT-TC-CC | 0.00001252 | TT-TC-CC | 0.0001811 | TT-TC-CC |
| rs1430961 | 90552920 | unknown | MMRN1 | 0.000004339 | TT-CT-CC | 0.001779 | TT-CC-CT | 0.0001479 | CC-TT-CT |
| rs2736990 | 90678541 | intron | SNCA | 0.000004604 | TT-CT-CC | 0.1164 | TT-CT-CC | 0.2426 | TT-CT-CC |
| rs356204 | 90663542 | intron | SNCA | 0.000004604 | GG-AG-AA | 0.1244 | GG-AG-AA | 0.2302 | GG-AG-AA |
| rs356168 | 90674431 | intron | SNCA | 0.000004604 | AA-GA-GG | 0.1376 | AA-GA-GG | 0.2302 | AA-GA-GG |
| rs356200 | 90668614 | intron | SNCA | 0.000004604 | GG-AG-AA | 0.1616 | GG-AG-AA | 0.2439 | GG-AG-AA |
| rs2737033 | 90707947 | intron | SNCA | 0.00000469 | AA-GA-GG | 0.09868 | AA-GA-GG | 0.2891 | AA-GA-GG |
| rs3775465 | 90827466 | intron | MMRN1 | 0.000004713 | AA-CA-CC | 6.338E-007 | AA-CA-CC | 0.00003808 | AA-CC-CA |

* Genotypes are ordered by mean expression values from the smaller to the higher.
