# Supplementary material for: Refining Susceptibility Loci of Chronic Obstructive Pulmonary Disease with Lung eqtls
Source: PLoS One. 2013 Jul 30;8(7):e70220. doi: 10.1371/journal.pone.0070220 (PMC3728203; doi:10.1371/journal.pone.0070220)
Supplement: Table S2 — Significant eQTLs at the 4q31 locus in the Laval dataset and replication in UBC and Groningen datasets. (DOCX) [file pone.0070220.s005.docx]

**Table S2: Significant eQTLs at the 4q31 locus in the Laval dataset and replication in UBC and Groningen datasets.**

| **SNP** | **SNP Position** | **SNP Function** | **Gene Regulated** | **P Laval** | **eQTL Direction Laval*** | **P UBC** | **eQTL Direction UBC*** | **P Groningen** | **eQTL Direction Groningen*** |
| --- | --- | --- | --- | --- | --- | --- | --- | --- | --- |
| rs12498677 | 146080339 | intron | OTUD4 | 1.058E-008 | CC-CT-TT | 0.0002261 | CC-CT-TT | 0.1305 | CT-TT |
| rs7667092 | 144889757 | unknown | BC029578 | 1.778E-008 | CC-CT-TT | 7.252E-010 | CC-CT-TT | 5.95E-011 | CC-CT-TT |
| rs6826862 | 144899333 | unknown | BC029578 | 3.379E-008 | AA-AG-GG | 7.252E-010 | AA-AG-GG | 5.95E-011 | AA-AG-GG |
| rs4287958 | 144908872 | unknown | BC029578 | 4.828E-008 | TT-TC-CC | 7.252E-010 | TT-TC-CC | 5.95E-011 | TT-TC-CC |
| rs1912745 | 144878737 | unknown | BC029578 | 7.393E-008 | AA-AG-GG | 2.313E-009 | AA-AG | 3.481E-011 | AA-AG-GG |
| rs10027246 | 144856222 | unknown | BC029578 | 1.507E-007 | CC-CA-AA | 0.000000025 | CC-CA-AA | 4.909E-011 | CC-CA-AA |
| rs1545437 | 144617842 | coding-synon | BC029578 | 1.648E-007 | TT-TG-GG | 3.375E-008 | TT-TG-GG | 7.691E-012 | TT-TG-GG |
| rs34714886 | 145020544 | unknown | BC029578 | 0.000000185 | CC-CT-TT | 2.154E-009 | CC-CT-TT | 3.59E-009 | CC-CT-TT |
| rs1580002 | 144687660 | unknown | BC029578 | 1.957E-007 | AA-AG-GG | 1.258E-008 | AA-AG-GG | 1.242E-011 | AA-AG-GG |
| rs9308176 | 144993871 | unknown | BC029578 | 1.994E-007 | GG-GA-AA | 9.242E-010 | GG-GA-AA | 1.078E-008 | GG-GA-AA |
| rs10030428 | 144683852 | unknown | BC029578 | 2.036E-007 | GG-GA-AA | 3.268E-008 | GG-GA-AA | 1.217E-011 | GG-GA-AA |
| rs35243309 | 144676269 | unknown | BC029578 | 2.065E-007 | CC-CT-TT | 9.29E-009 | CC-CT-TT | 9.707E-012 | CC-CT-TT |
| rs4835305 | 144744799 | unknown | BC029578 | 2.203E-007 | AA-AG-GG | 5.569E-009 | AA-AG-GG | 9.707E-012 | AA-AG-GG |
| rs13105102 | 144740199 | unknown | BC029578 | 2.203E-007 | AA-AG-GG | 1.258E-008 | AA-AG-GG | 1.242E-011 | AA-AG-GG |
| rs6828935 | 144750266 | unknown | BC029578 | 2.401E-007 | CC-CT-TT | 3.648E-009 | CC-CT-TT | 3.497E-012 | CC-CT-TT |
| rs7681914 | 144629991 | unknown | BC029578 | 2.469E-007 | AA-AG-GG | 5.193E-008 | AA-AG-GG | 6.54E-012 | AA-AG-GG |
| rs924234 | 144627952 | unknown | BC029578 | 2.469E-007 | CC-CA-AA | 5.193E-008 | CC-CA-AA | 6.54E-012 | CC-CA-AA |
| rs9685306 | 144669996 | unknown | BC029578 | 0.000000257 | AA-AG-GG | 6.83E-008 | AA-AG-GG | 5.973E-012 | AA-AG-GG |
| rs1822841 | 144842638 | unknown | BC029578 | 2.837E-007 | GG-GT-TT | 2.541E-008 | GG-GT-TT | 9.62E-011 | GG-GT-TT |
| rs10857415 | 144829906 | unknown | BC029578 | 0.000000285 | CC-CT-TT | 4.485E-008 | CC-CT-TT | 9.62E-011 | CC-CT-TT |
| rs9997931 | 144825754 | intron | BC029578 | 2.973E-007 | TT-TC-CC | 8.884E-009 | TT-TC-CC | 7.635E-011 | TT-TC-CC |
| rs4240334 | 144792505 | untranslated-3 | BC029578 | 2.973E-007 | GG-GA-AA | 1.992E-008 | GG-GA-AA | 2.231E-011 | GG-GA-AA |
| rs7687915 | 144680954 | unknown | BC029578 | 3.114E-007 | GG-GT-TT | 8.701E-009 | GG-GT-TT | 1.748E-011 | GG-GT-TT |
| rs12512202 | 144692401 | unknown | BC029578 | 3.217E-007 | GG-GA-AA | 5.569E-009 | GG-GA-AA | 9.707E-012 | GG-GA-AA |
| rs13129702 | 144706285 | unknown | BC029578 | 3.217E-007 | CC-CT-TT | 5.569E-009 | CC-CT-TT | 9.707E-012 | CC-CT-TT |
| rs1597445 | 144689696 | unknown | BC029578 | 3.217E-007 | GG-GA-AA | 5.569E-009 | GG-GA-AA | 9.707E-012 | GG-GA-AA |
| rs6537183 | 144691594 | unknown | BC029578 | 3.217E-007 | GG-GA-AA | 5.569E-009 | GG-GA-AA | 9.707E-012 | GG-GA-AA |
| rs10016223 | 144698582 | unknown | BC029578 | 3.217E-007 | GG-GA-AA | 5.569E-009 | GG-GA-AA | 1.203E-011 | GG-GA-AA |
| rs4383567 | 144710026 | unknown | BC029578 | 3.217E-007 | GG-GA-AA | 6.25E-009 | GG-GA-AA | 9.707E-012 | GG-GA-AA |
| rs1006406 | 144682863 | unknown | BC029578 | 3.217E-007 | AA-AG-GG | 9.29E-009 | AA-AG-GG | 9.707E-012 | AA-AG-GG |
| rs13134327 | 144659795 | unknown | BC029578 | 3.217E-007 | AA-AG-GG | 9.29E-009 | AA-AG-GG | 9.707E-012 | AA-AG-GG |
| rs1375984 | 144681663 | unknown | BC029578 | 3.217E-007 | TT-TG-GG | 9.29E-009 | TT-TG-GG | 9.707E-012 | TT-TG-GG |
| rs2323190 | 144665435 | unknown | BC029578 | 3.217E-007 | TT-TC-CC | 9.29E-009 | TT-TC-CC | 9.707E-012 | TT-TC-CC |
| rs1375989 | 144648046 | unknown | BC029578 | 3.217E-007 | GG-GT-TT | 0.000000021 | GG-GT-TT | 1.242E-011 | GG-GT-TT |
| rs1450250 | 144653026 | unknown | BC029578 | 3.217E-007 | AA-AG-GG | 0.000000021 | AA-AG-GG | 1.242E-011 | AA-AG-GG |
| rs1849136 | 144833533 | unknown | BC029578 | 0.00000054 | AA-AG-GG | 1.992E-008 | AA-AG-GG | 9.62E-011 | AA-AG-GG |
| rs7655625 | 145485915 | unknown | HHIP | 6.687E-007 | CC-CT-TT | 0.01043 | CC-CT-TT | 0.2136 | CC-CT-TT |
| rs1489759 | 145474473 | unknown | HHIP | 6.687E-007 | CC-CT-TT | 0.01284 | CC-CT-TT | 0.2136 | CC-CT-TT |
| rs1828591 | 145480780 | unknown | HHIP | 6.687E-007 | GG-GA-AA | 0.01284 | GG-GA-AA | 0.2136 | GG-GA-AA |
| rs1375982 | 144666917 | unknown | BC029578 | 8.758E-007 | GG-GA-AA | 2.376E-008 | GG-GA-AA | 1.361E-011 | GG-GA-AA |
| rs6844670 | 145024799 | unknown | BC029578 | 9.383E-007 | GG-GA-AA | 1.247E-007 | GG-GA-AA | 0.000001696 | GG-GA-AA |
| rs1512282 | 145431497 | unknown | HHIP | 0.000001085 | CC-CT-TT | 0.03421 | CC-CT-TT | 0.6693 | CC-CT-TT |
| rs13118928 | 145486389 | unknown | HHIP | 0.000001167 | GG-GA-AA | 0.01043 | GG-GA-AA | 0.2121 | GG-GA-AA |
| rs1980057 | 145485738 | unknown | HHIP | 0.000001167 | TT-TC-CC | 0.01043 | TT-TC-CC | 0.2136 | TT-TC-CC |
| rs11938745 | 145465768 | unknown | HHIP | 0.000001405 | CC-CT-TT | 0.00962 | CC-CT-TT | 0.2136 | CC-CT-TT |
| rs720485 | 145462588 | unknown | HHIP | 0.000001405 | CC-CA-AA | 0.01043 | CC-CA-AA | 0.2108 | CC-CA-AA |
| rs6828540 | 145463231 | unknown | HHIP | 0.000001405 | AA-AG-GG | 0.01043 | AA-AG-GG | 0.2136 | AA-AG-GG |
| rs7655625 | 145485915 | unknown | HHIP | 0.000002097 | CC-CT-TT | 0.2619 | CC-TT-CT | 0.2692 | CC-CT-TT |
| rs1489759 | 145474473 | unknown | HHIP | 0.000002097 | CC-CT-TT | 0.3051 | CC-TT-CT | 0.2692 | CC-CT-TT |
| rs1828591 | 145480780 | unknown | HHIP | 0.000002097 | GG-GA-AA | 0.3051 | GG-AA-GA | 0.2692 | GG-GA-AA |
| rs9799404 | 144894260 | unknown | BC029578 | 0.000002112 | TT-CT-CC | 2.918E-007 | TT-CT-CC | 0.000003758 | TT-CT-CC |
| rs7676614 | 144616366 | intron | BC029578 | 0.000002168 | GG-GA-AA | 5.218E-008 | GG-GA-AA | 7.461E-012 | GG-GA-AA |
| rs7657795 | 145009802 | unknown | BC029578 | 0.000002281 | CC-AC-AA | 0.0002836 | CC-AC-AA | 0.01917 | CC-AC-AA |
| rs7679112 | 145009868 | unknown | BC029578 | 0.000002641 | AA-GA-GG | 0.0002836 | AA-GA-GG | 0.01917 | AA-GA-GG |
| rs1512288 | 145491281 | unknown | HHIP | 0.000003 | TT-TC-CC | 0.009181 | TT-TC-CC | 0.2272 | TT-TC-CC |
| rs6817273 | 145492003 | unknown | HHIP | 0.000003 | CC-CT-TT | 0.009181 | CC-CT-TT | 0.2272 | CC-CT-TT |
| rs1980057 | 145485738 | unknown | HHIP | 0.000003207 | TT-TC-CC | 0.2619 | TT-CC-TC | 0.2692 | TT-TC-CC |
| rs13118928 | 145486389 | unknown | HHIP | 0.000003207 | GG-GA-AA | 0.2619 | GG-AA-GA | 0.2714 | GG-GA-AA |
| rs13142879 | 145410477 | unknown | HHIP | 0.000004015 | TT-TC-CC | 0.0622 | TT-TC-CC | 0.754 | TT-CC-TC |
| rs11938745 | 145465768 | unknown | HHIP | 0.000004283 | CC-CT-TT | 0.2408 | CC-TT-CT | 0.2692 | CC-CT-TT |
| rs6828540 | 145463231 | unknown | HHIP | 0.000004283 | AA-AG-GG | 0.2619 | AA-GG-AG | 0.2692 | AA-AG-GG |
| rs720485 | 145462588 | unknown | HHIP | 0.000004283 | CC-CA-AA | 0.2619 | CC-AA-CA | 0.299 | CC-CA-AA |
| rs7687915 | 144680954 | unknown | FREM3 | 0.000005207 | TT-GT-GG | 0.001205 | TT-GT-GG | 0.247 | TT-GT-GG |
| rs6828935 | 144750266 | unknown | FREM3 | 0.00000566 | TT-CT-CC | 0.0007205 | TT-CT-CC | 0.4396 | TT-CC-CT |
| rs34714886 | 145020544 | unknown | FREM3 | 0.000006424 | TT-CT-CC | 0.001255 | TT-CT-CC | 0.5159 | CC-TT-CT |
| rs10030428 | 144683852 | unknown | FREM3 | 0.000006985 | AA-GA-GG | 0.00135 | AA-GA-GG | 0.4564 | AA-GG-GA |
| rs1512288 | 145491281 | unknown | HHIP | 0.000007249 | TT-TC-CC | 0.2221 | TT-CC-TC | 0.2359 | TT-TC-CC |
| rs6817273 | 145492003 | unknown | HHIP | 0.000007249 | CC-CT-TT | 0.2221 | CC-TT-CT | 0.2359 | CC-CT-TT |
| rs1849136 | 144833533 | unknown | FREM3 | 0.000007624 | GG-AG-AA | 0.000158 | GG-AG-AA | 0.3733 | GG-AA-AG |
| rs6828935 | 144750266 | unknown | FREM3 | 0.000007857 | TT-CT-CC | 0.001121 | TT-CT-CC | 0.2769 | TT-CT-CC |
| rs4240334 | 144792505 | untranslated-3 | FREM3 | 0.000008335 | AA-GA-GG | 0.000158 | AA-GA-GG | 0.3727 | AA-GG-GA |
| rs9997931 | 144825754 | intron | FREM3 | 0.000008335 | CC-TC-TT | 0.0006703 | CC-TC-TT | 0.4117 | CC-TT-TC |
| rs10027246 | 144856222 | unknown | FREM3 | 0.000008531 | AA-CA-CC | 0.0001903 | AA-CA-CC | 0.3679 | AA-CC-CA |
| rs1375982 | 144666917 | unknown | FREM3 | 0.000008601 | AA-GA-GG | 0.00132 | AA-GA-GG | 0.3928 | AA-GG-GA |
| rs13105102 | 144740199 | unknown | FREM3 | 0.000008752 | GG-AG-AA | 0.0001986 | GG-AG-AA | 0.4093 | GG-AA-AG |
| rs4835305 | 144744799 | unknown | FREM3 | 0.000008752 | GG-AG-AA | 0.0008227 | GG-AG-AA | 0.4496 | GG-AA-AG |
| rs34330278 | 146037320 | intron | OTUD4 | 0.000009176 | AA-AG-GG | 0.002125 | AA-AG-GG | 0.01447 | AG-GG |
| rs1375982 | 144666917 | unknown | FREM3 | 0.0000102 | AA-GA-GG | 0.001758 | AA-GA-GG | 0.3005 | AA-GA-GG |
| rs1375989 | 144648046 | unknown | FREM3 | 0.00001042 | TT-GT-GG | 0.0002881 | TT-GT-GG | 0.4093 | TT-GG-GT |
| rs1450250 | 144653026 | unknown | FREM3 | 0.00001042 | GG-AG-AA | 0.0002881 | GG-AG-AA | 0.4093 | GG-AA-AG |
| rs4383567 | 144710026 | unknown | FREM3 | 0.00001042 | AA-GA-GG | 0.000744 | AA-GA-GG | 0.4496 | AA-GG-GA |
| rs12512202 | 144692401 | unknown | FREM3 | 0.00001042 | AA-GA-GG | 0.0008227 | AA-GA-GG | 0.4496 | AA-GG-GA |
| rs13129702 | 144706285 | unknown | FREM3 | 0.00001042 | TT-CT-CC | 0.0008227 | TT-CT-CC | 0.4496 | TT-CC-CT |
| rs1597445 | 144689696 | unknown | FREM3 | 0.00001042 | AA-GA-GG | 0.0008227 | AA-GA-GG | 0.4496 | AA-GG-GA |
| rs6537183 | 144691594 | unknown | FREM3 | 0.00001042 | AA-GA-GG | 0.0008227 | AA-GA-GG | 0.4496 | AA-GG-GA |
| rs10016223 | 144698582 | unknown | FREM3 | 0.00001042 | AA-GA-GG | 0.0008227 | AA-GA-GG | 0.45 | AA-GG-GA |
| rs1006406 | 144682863 | unknown | FREM3 | 0.00001042 | GG-AG-AA | 0.001146 | GG-AG-AA | 0.4496 | GG-AA-AG |
| rs13134327 | 144659795 | unknown | FREM3 | 0.00001042 | GG-AG-AA | 0.001146 | GG-AG-AA | 0.4496 | GG-AA-AG |
| rs1375984 | 144681663 | unknown | FREM3 | 0.00001042 | GG-TG-TT | 0.001146 | GG-TG-TT | 0.4496 | GG-TT-TG |
| rs2323190 | 144665435 | unknown | FREM3 | 0.00001042 | CC-TC-TT | 0.001146 | CC-TC-TT | 0.4496 | CC-TT-TC |
| rs4240334 | 144792505 | untranslated-3 | FREM3 | 0.00001066 | AA-GA-GG | 0.000346 | AA-GA-GG | 0.2063 | AA-GA-GG |
| rs9997931 | 144825754 | intron | FREM3 | 0.00001066 | CC-TC-TT | 0.001133 | CC-TC-TT | 0.195 | CC-TC-TT |
| rs9685306 | 144669996 | unknown | FREM3 | 0.00001072 | GG-AG-AA | 0.0003419 | GG-AG-AA | 0.3779 | GG-AA-AG |
| rs10857415 | 144829906 | unknown | FREM3 | 0.00001128 | TT-CT-CC | 0.0002189 | TT-CT-CC | 0.3733 | TT-CC-CT |
| rs9308176 | 144993871 | unknown | FREM3 | 0.00001135 | AA-GA-GG | 0.003739 | AA-GA-GG | 0.5005 | AA-GG-GA |
| rs10027246 | 144856222 | unknown | FREM3 | 0.00001142 | AA-CA-CC | 0.0003801 | AA-CA-CC | 0.1791 | AA-CA-CC |
| rs13105102 | 144740199 | unknown | FREM3 | 0.00001372 | GG-AG-AA | 0.0003398 | GG-AG-AA | 0.2147 | GG-AG-AA |
| rs4835305 | 144744799 | unknown | FREM3 | 0.00001372 | GG-AG-AA | 0.001117 | GG-AG-AA | 0.2403 | GG-AG-AA |
| rs9685306 | 144669996 | unknown | FREM3 | 0.00001373 | GG-AG-AA | 0.0003246 | GG-AG-AA | 0.2567 | GG-AG-AA |
| rs1375989 | 144648046 | unknown | FREM3 | 0.00001423 | TT-GT-GG | 0.0003781 | TT-GT-GG | 0.2147 | TT-GT-GG |
| rs1450250 | 144653026 | unknown | FREM3 | 0.00001423 | GG-AG-AA | 0.0003781 | GG-AG-AA | 0.2147 | GG-AG-AA |
| rs4383567 | 144710026 | unknown | FREM3 | 0.00001423 | AA-GA-GG | 0.001103 | AA-GA-GG | 0.2403 | AA-GA-GG |
| rs12512202 | 144692401 | unknown | FREM3 | 0.00001423 | AA-GA-GG | 0.001117 | AA-GA-GG | 0.2403 | AA-GA-GG |
| rs13129702 | 144706285 | unknown | FREM3 | 0.00001423 | TT-CT-CC | 0.001117 | TT-CT-CC | 0.2403 | TT-CT-CC |
| rs1597445 | 144689696 | unknown | FREM3 | 0.00001423 | AA-GA-GG | 0.001117 | AA-GA-GG | 0.2403 | AA-GA-GG |
| rs6537183 | 144691594 | unknown | FREM3 | 0.00001423 | AA-GA-GG | 0.001117 | AA-GA-GG | 0.2403 | AA-GA-GG |
| rs10016223 | 144698582 | unknown | FREM3 | 0.00001423 | AA-GA-GG | 0.001117 | AA-GA-GG | 0.2491 | AA-GA-GG |
| rs1006406 | 144682863 | unknown | FREM3 | 0.00001423 | GG-AG-AA | 0.001231 | GG-AG-AA | 0.2403 | GG-AG-AA |
| rs13134327 | 144659795 | unknown | FREM3 | 0.00001423 | GG-AG-AA | 0.001231 | GG-AG-AA | 0.2403 | GG-AG-AA |
| rs1375984 | 144681663 | unknown | FREM3 | 0.00001423 | GG-TG-TT | 0.001231 | GG-TG-TT | 0.2403 | GG-TG-TT |
| rs2323190 | 144665435 | unknown | FREM3 | 0.00001423 | CC-TC-TT | 0.001231 | CC-TC-TT | 0.2403 | CC-TC-TT |
| rs1822841 | 144842638 | unknown | FREM3 | 0.00001426 | TT-GT-GG | 0.0001058 | TT-GT-GG | 0.3733 | TT-GG-GT |

* Genotypes are ordered by mean expression values from the smaller to the higher.
