# Supplementary material for: Refining Susceptibility Loci of Chronic Obstructive Pulmonary Disease with Lung eqtls
Source: PLoS One. 2013 Jul 30;8(7):e70220. doi: 10.1371/journal.pone.0070220 (PMC3728203; doi:10.1371/journal.pone.0070220)
Supplement: Table S3 — Significant eQTLs at the 19q13 locus in the Laval dataset and replication in UBC and Groningen datasets. (DOCX) [file pone.0070220.s006.docx]

**Table S3: Significant eQTLs at the 19q13 locus in the Laval dataset and replication in UBC and Groningen datasets.**

| **SNP** | **SNP Position** | **SNP Function** | **Regulated Gene** | **P Laval** | **eQTL Direction Laval*** | **P UBC** | **eQTL Direction UBC*** | **P Groningen** | **eQTL Direction Groningen*** |
| --- | --- | --- | --- | --- | --- | --- | --- | --- | --- |
| rs2191139 | 42001210 | unknown | LOC100505495 | 9.721E-082 | TT-TC-CC | 3.223E-067 | TT-TC-CC | 1.134E-071 | TT-TC-CC |
| rs758643 | 41991524 | unknown | LOC100505495 | 3.95E-056 | TT-TC-CC | 2.622E-041 | TT-TC-CC | 2.483E-049 | TT-TC-CC |
| rs11672691 | 41985587 | unknown | LOC100505495 | 7.103E-055 | AA-AG-GG | 9.861E-056 | AA-AG-GG | 7.238E-054 | AA-AG-GG |
| rs2302188 | 42085873 | missense | CEACAM21 | 7.741E-051 | AA-AG-GG | 8.865E-030 | AA-AG-GG | 3.361E-021 | AA-AG-GG |
| rs887391 | 41985624 | unknown | LOC100505495 | 2.09E-049 | CC-CT-TT | 4.203E-040 | CC-CT-TT | 2.007E-046 | CC-CT-TT |
| rs714106 | 42083849 | missense | CEACAM21 | 1.074E-045 | CC-CA-AA | 3.146E-027 | CC-CA-AA | 1.23E-019 | CC-CA-AA |
| rs4803481 | 42066556 | unknown | CEACAM21 | 1.231E-045 | AA-AG-GG | 1.217E-027 | AA-AG-GG | 3.597E-019 | AA-AG-GG |
| rs28605876 | 41931454 | untranslated-3 | B3GNT8 | 1.718E-040 | GG-AG-AA | 1.968E-012 | GG-AG-AA | 5.115E-014 | GG-AG-AA |
| rs284662 | 41932275 | missense | B3GNT8 | 4.694E-039 | GG-AG-AA | 2.966E-011 | GG-AG-AA | 2.806E-014 | GG-AG-AA |
| rs10409485 | 41920765 | intron | B3GNT8 | 2E-038 | CC-TC-TT | 1.514E-011 | CC-TC-TT | 5.693E-015 | CC-TC-TT |
| rs1473248 | 41923314 | intron | B3GNT8 | 2E-038 | GG-AG-AA | 1.514E-011 | GG-AG-AA | 5.693E-015 | GG-AG-AA |
| rs284652 | 41928652 | coding-synon | B3GNT8 | 2E-038 | AA-GA-GG | 1.514E-011 | AA-GA-GG | 5.693E-015 | AA-GA-GG |
| rs4674 | 41930396 | coding-synon | B3GNT8 | 2E-038 | CC-TC-TT | 2.884E-011 | CC-TC-TT | 7.821E-014 | CC-TC-TT |
| rs284663 | 41932612 | coding-synon | B3GNT8 | 2E-038 | AA-GA-GG | 3.063E-011 | AA-GA-GG | 4.925E-014 | AA-GA-GG |
| rs4803465 | 41918158 | intron | B3GNT8 | 2.775E-038 | TT-CT-CC | 1.514E-011 | TT-CT-CC | 5.693E-015 | TT-CT-CC |
| rs11880539 | 41918949 | intron | B3GNT8 | 2.775E-038 | AA-CA-CC | 1.514E-011 | AA-CA-CC | 8.1E-014 | AA-CA-CC |
| rs11670757 | 41913431 | intron | B3GNT8 | 2.775E-038 | TT-GT-GG | 2.733E-011 | TT-GT-GG | 4.121E-014 | TT-GT-GG |
| rs284661 | 41932120 | coding-synon | B3GNT8 | 2.775E-038 | AA-GA-GG | 3.063E-011 | AA-GA-GG | 6.297E-013 | AA-GA-GG |
| rs12973063 | 42009673 | unknown | LOC100505495 | 1.079E-037 | GG-GA-AA | 5.485E-036 | GG-GA-AA | 2.725E-051 | GG-GA-AA |
| rs11882144 | 42072602 | unknown | CEACAM21 | 1.267E-037 | GG-GA-AA | 7.011E-035 | GG-GA-AA | 8.253E-019 | GG-GA-AA |
| rs4803480 | 42066279 | unknown | CEACAM21 | 1.267E-037 | AA-AG-GG | 4.452E-033 | AA-AG-GG | 4.601E-018 | AA-AG-GG |
| rs2231940 | 41944237 | missense | B3GNT8 | 1.605E-037 | AA-GA-GG | 2.212E-011 | AA-GA-GG | 1.697E-012 | AA-GA-GG |
| rs9304593 | 41942875 | intron | B3GNT8 | 2.211E-037 | AA-CA-CC | 2.212E-011 | AA-CA-CC | 3.081E-012 | AA-CA-CC |
| rs13344547 | 41945848 | near-gene-5 | B3GNT8 | 2.211E-037 | GG-TG-TT | 2.365E-011 | GG-TG-TT | 1.697E-012 | GG-TG-TT |
| rs3745290 | 41890003 | near-gene-3 | B3GNT8 | 4.201E-037 | AA-GA-GG | 4.73E-012 | AA-GA-GG | 2.637E-014 | AA-GA-GG |
| rs878082 | 41897674 | intron | B3GNT8 | 4.201E-037 | AA-GA-GG | 8.013E-012 | AA-GA-GG | 2.637E-014 | AA-GA-GG |
| rs10853751 | 41903220 | missense | B3GNT8 | 4.354E-037 | TT-CT-CC | 9.723E-012 | TT-CT-CC | 1.776E-015 | TT-CT-CC |
| rs3810174 | 41904165 | intron | B3GNT8 | 4.354E-037 | TT-CT-CC | 9.723E-012 | TT-CT-CC | 1.776E-015 | TT-CT-CC |
| rs892044 | 41903671 | near-gene-5 | B3GNT8 | 4.354E-037 | CC-TC-TT | 9.723E-012 | CC-TC-TT | 1.776E-015 | CC-TC-TT |
| rs12602 | 41889748 | coding-synon | B3GNT8 | 1.341E-036 | GG-AG-AA | 3.604E-011 | GG-AG-AA | 2.669E-015 | GG-AG-AA |
| rs4141218 | 42081907 | near-gene-5 | CEACAM21 | 5.406E-034 | TT-TC-CC | 3.042E-031 | TT-TC-CC | 2.283E-017 | TT-TC-CC |
| rs17318596 | 41937095 | near-gene-3 | B3GNT8 | 3.102E-033 | GG-AG-AA | 2.52E-009 | GG-AG-AA | 1.462E-013 | GG-AG-AA |
| rs4802122 | 42047924 | unknown | CEACAM21 | 2.588E-028 | GG-GT-TT | 5.989E-022 | GG-GT-TT | 8.856E-011 | GG-GT-TT |
| rs7255742 | 42049821 | unknown | CEACAM21 | 4.744E-028 | AA-AG-GG | 1.338E-024 | AA-AG-GG | 2.695E-012 | AA-AG-GG |
| rs10412700 | 42020942 | unknown | LOC100505495 | 2.305E-026 | TT-TC-CC | 4.7E-034 | TT-TC-CC | 1.592E-038 | TC-CC |
| rs12978323 | 41756038 | intron | AXL | 3.761E-026 | TT-GT-GG | 4.904E-011 | TT-GT-GG | 5.776E-008 | TT-GT-GG |
| rs12980267 | 41765229 | intron | AXL | 6.755E-026 | TT-CT-CC | 8.064E-009 | TT-CT-CC | 5.333E-008 | TT-CT-CC |
| rs1946612 | 41767987 | near-gene-3 | AXL | 6.755E-026 | CC-TC-TT | 8.064E-009 | CC-TC-TT | 5.333E-008 | CC-TC-TT |
| rs7250883 | 41760033 | intron | AXL | 6.755E-026 | TT-CT-CC | 8.064E-009 | TT-CT-CC | 5.333E-008 | TT-CT-CC |
| rs3760659 | 41769104 | intron | AXL | 6.755E-026 | TT-GT-GG | 8.064E-009 | TT-GT-GG | 6.769E-008 | TT-GT-GG |
| rs371500 | 40480923 | intron | ZNF780A | 5.235E-025 | CC-TT-TC | 3.765E-014 | CC-TT-TC | 3.563E-010 | CC-TC |
| rs337799 | 40591182 | intron | ZNF780A | 2.072E-024 | TT-CT-CC | 1.53E-021 | TT-CT-CC | 2.834E-016 | TT-CT-CC |
| rs16974093 | 40650002 | unknown | ZNF780A | 2.072E-024 | GG-AG-AA | 3.029E-020 | GG-AG-AA | 1.653E-016 | GG-AG-AA |
| rs10402038 | 40637615 | unknown | ZNF780A | 2.072E-024 | TT-CT-CC | 3.029E-020 | TT-CT-CC | 2.834E-016 | TT-CT-CC |
| rs3826716 | 42035716 | unknown | LOC100505495 | 3.751E-024 | AG-AA-GG | 4.348E-032 | AA-AG-GG | 2.16E-030 | AA-AG-GG |
| rs11668699 | 42031592 | unknown | LOC100505495 | 3.751E-024 | AT-AA-TT | 1.647E-030 | AA-AT-TT | 5.135E-031 | AA-AT-TT |
| rs3826716 | 42035716 | unknown | CEACAM21 | 6.066E-024 | AA-AG-GG | 2.575E-018 | AA-AG-GG | 3.657E-009 | AA-AG-GG |
| rs11668699 | 42031592 | unknown | CEACAM21 | 6.066E-024 | AA-AT-TT | 1.204E-017 | AA-AT-TT | 0.000000002 | AA-AT-TT |
| rs12980480 | 41804655 | intron | AXL | 9.161E-024 | AA-GA-GG | 1.321E-013 | AA-GA-GG | 6.316E-010 | AA-GA-GG |
| rs2304230 | 41774064 | intron | AXL | 9.161E-024 | AA-GA-GG | 1.321E-013 | AA-GA-GG | 6.316E-010 | AA-GA-GG |
| rs3745295 | 41785282 | intron | AXL | 9.161E-024 | AA-CA-CC | 1.321E-013 | AA-CA-CC | 6.316E-010 | AA-CA-CC |
| rs3752705 | 41798111 | intron | AXL | 9.161E-024 | TT-CT-CC | 1.321E-013 | TT-CT-CC | 6.316E-010 | TT-CT-CC |
| rs3816052 | 41773797 | intron | AXL | 9.161E-024 | CC-TC-TT | 1.321E-013 | CC-TC-TT | 2.608E-009 | CC-TC-TT |
| rs7248164 | 41807700 | intron | AXL | 9.161E-024 | GG-AG-AA | 1.321E-013 | GG-AG-AA | 2.608E-009 | GG-AG-AA |
| rs2304231 | 41762670 | intron | AXL | 1.301E-023 | AA-GA-GG | 1.1E-011 | AA-GA-GG | 2.029E-008 | AA-GA-GG |
| rs2304232 | 41762525 | intron | AXL | 1.301E-023 | TT-CT-CC | 1.1E-011 | TT-CT-CC | 2.63E-008 | TT-CT-CC |
| rs4591267 | 41765407 | intron | AXL | 1.9E-023 | AA-CA | 1.147E-010 | AA-CA-CC | 5.709E-009 | AA-CA-CC |
| rs11879090 | 41917260 | intron | B3GNT8 | 3.103E-023 | TT-TC-CC | 1.306E-009 | TT-TC-CC | 9.903E-012 | TT-TC-CC |
| rs284653 | 41928701 | intron | B3GNT8 | 3.103E-023 | AA-AG-GG | 1.408E-009 | AA-AG-GG | 9.903E-012 | AA-AG-GG |
| rs11879435 | 41757603 | intron | AXL | 4.748E-023 | CC-TC-TT | 7.53E-012 | CC-TC-TT | 2.665E-007 | CC-TC-TT |
| rs459091 | 40561175 | intron | ZNF780A | 5.342E-023 | AA-GA-GG | 5.513E-014 | AA-GA-GG | 1.442E-010 | AA-GA |
| rs426950 | 40562386 | near-gene-5 | ZNF780A | 5.784E-023 | TT-CT-CC | 5.728E-015 | TT-CT-CC | 1.442E-010 | TT-CT |
| rs421533 | 40579468 | intron | ZNF780A | 5.784E-023 | CC-TC-TT | 3.64E-014 | CC-TC-TT | 1.442E-010 | CC-TC |
| rs234370 | 40477465 | intron | ZNF780A | 5.784E-023 | AA-GA-GG | 5.513E-014 | AA-GA-GG | 1.442E-010 | AA-GA |
| rs8105066 | 40489470 | unknown | ZNF780A | 5.784E-023 | GG-AG-AA | 5.513E-014 | GG-AG-AA | 1.442E-010 | GG-AG |
| rs234346 | 40467676 | unknown | ZNF780A | 5.784E-023 | TT-CT-CC | 5.513E-014 | TT-CT-CC | 2.669E-010 | TT-CT |
| rs11882467 | 41754217 | intron | AXL | 7.435E-023 | GG-TG-TT | 1.561E-008 | GG-TG-TT | 1.997E-007 | GG-TG-TT |
| rs170901 | 40532800 | unknown | ZNF780A | 7.923E-023 | GG-AA-AG | 1.56E-015 | GG-AA-AG | 8.257E-011 | GG-AG |
| rs12974420 | 41798660 | intron | AXL | 8.448E-023 | AA-GA-GG | 6.973E-013 | AA-GA-GG | 6.316E-010 | AA-GA-GG |
| rs4577216 | 40569551 | unknown | ZNF780A | 9.677E-023 | TT-CC-CT | 4.808E-016 | TT-CC-CT | 1.292E-010 | TT-CT |
| rs24136 | 40596207 | intron | ZNF780A | 1.05E-022 | TT-CC-CT | 4.059E-014 | TT-CC-CT | 1.292E-010 | TT-CT |
| rs337789 | 40599603 | unknown | ZNF780A | 1.05E-022 | CC-AA-AC | 4.059E-014 | CC-AA-AC | 1.292E-010 | CC-AC |
| rs1327189 | 40627509 | unknown | ZNF780A | 4.12E-022 | GG-TG-TT | 6.717E-012 | GG-TT-TG | 2.796E-009 | GG-TG |
| rs17761603 | 40641794 | unknown | ZNF780A | 4.12E-022 | TT-CT-CC | 6.717E-012 | TT-CC-CT | 2.796E-009 | TT-CT |
| rs1998931 | 40628064 | unknown | ZNF780A | 4.12E-022 | CC-AC-AA | 5.33E-011 | CC-AA-AC | 2.796E-009 | CC-AC |
| rs2767607 | 40611153 | unknown | ZNF780A | 4.747E-022 | TT-CT-CC | 6.206E-012 | TT-CT | 2.796E-009 | TT-CT |
| GA016366 | NA | NA | ZNF780A | 4.783E-022 | CC-TC | 1.821E-013 | CC-TT-TC | 2.796E-009 | CC-TC |
| rs234324 | 40502919 | near-gene-5 | ZNF780A | 4.952E-022 | AA-GA | 2.45E-013 | AA-GG-GA | 0.000000002 | AA-GA |
| rs183504 | 40508115 | intron | ZNF780A | 6.459E-022 | AA-GA-GG | 2.45E-013 | AA-GG-GA | 2.796E-009 | AA-GA |
| rs2767604 | 40609975 | unknown | ZNF780A | 6.459E-022 | TT-CT-CC | 6.717E-012 | TT-CC-CT | 2.796E-009 | TT-CT |
| rs2191241 | 42103552 | unknown | LOC100505495 | 2.745E-021 | GG-GA-AA | 1.313E-012 | GG-GA-AA | 9.883E-019 | GG-GA-AA |
| rs234325 | 40502943 | untranslated-5 | ZNF780A | 7.254E-021 | AA-CA | 2.621E-013 | AA-CC-CA | 0.000000002 | AA-CA |
| rs10412700 | 42020942 | unknown | CEACAM21 | 1.285E-020 | TT-TC-CC | 4.904E-017 | TT-TC-CC | 1.291E-008 | TC-CC |
| rs1056854 | 41809995 | coding-synon | AXL | 1.36E-020 | AA-GA-GG | 2.24E-012 | AA-GA-GG | 1.111E-009 | AA-GA-GG |
| rs1076362 | 42091163 | intron | CEACAM21 | 5.545E-020 | TT-TC-CC | 3.808E-009 | TT-TC-CC | 4.114E-007 | TT-TC-CC |
| rs1865053 | 41814412 | near-gene-5 | AXL | 6.876E-020 | AA-GA-GG | 3.623E-011 | AA-GA-GG | 1.206E-008 | AA-GA-GG |
| rs7245398 | 41810644 | intron | AXL | 9.23E-020 | AA-GA-GG | 3.623E-011 | AA-GA-GG | 0.00000005 | AA-GA-GG |
| rs234309 | 40517991 | intron | ZNF780A | 1.256E-019 | CC-AC-AA | 1.853E-012 | CC-AC-AA | 6.705E-009 | CC-AC |
| rs234353 | 40529219 | unknown | ZNF780A | 1.369E-019 | TT-CT | 1.853E-012 | TT-CT-CC | 6.705E-009 | TT-CT |
| rs2191241 | 42103552 | unknown | CEACAM21 | 1.474E-019 | GG-GA-AA | 1.531E-016 | GG-GA-AA | 2.791E-010 | GG-GA-AA |
| rs4802122 | 42047924 | unknown | LOC100505495 | 2.764E-019 | GT-GG-TT | 2.995E-024 | GG-GT-TT | 2.649E-023 | GG-GT-TT |
| rs12984160 | 41815713 | near-gene-5 | AXL | 6.605E-019 | AA-GA-GG | 1.639E-010 | AA-GA-GG | 1.206E-008 | AA-GA-GG |
| rs2005832 | 41983822 | unknown | LOC100505495 | 9.981E-019 | CC-CT-TT | 1.756E-032 | CC-CT-TT | 1.17E-018 | CC-CT-TT |
| rs12981943 | 42019187 | unknown | LOC100505495 | 1.436E-018 | GG-GA-AA | 1.521E-018 | GG-GA-AA | 7.315E-018 | GG-GA-AA |
| rs12610088 | 42020112 | unknown | LOC100505495 | 4.924E-018 | CC-CT-TT | 2.595E-018 | CC-CT-TT | 5.463E-018 | CC-CT-TT |
| GA034631 | NA | NA | AXL | 6.422E-018 | TT-AT-AA | 2.192E-009 | TT-AT-AA | 2.908E-008 | TT-AT-AA |
| rs2241718 | 41829606 | untranslated-3 | AXL | 8.234E-018 | CC-TC-TT | 1.469E-009 | CC-TC-TT | 0.000000013 | CC-TC-TT |
| rs714106 | 42083849 | missense | LOC100505495 | 1.045E-017 | CC-CA-AA | 5.617E-020 | CC-CA-AA | 8.316E-018 | CC-CA-AA |
| rs4803481 | 42066556 | unknown | LOC100505495 | 1.321E-017 | AA-AG-GG | 4.637E-020 | AA-AG-GG | 8.736E-019 | AA-AG-GG |
| rs12985162 | 41831507 | unknown | AXL | 1.428E-017 | GG-AG-AA | 4.331E-009 | GG-AG-AA | 2.845E-008 | GG-AG-AA |
| rs7257310 | 41827120 | intron | AXL | 2.371E-017 | CC-TC-TT | 1.921E-010 | CC-TC-TT | 1.206E-008 | CC-TC-TT |
| rs1865051 | 41824885 | intron | AXL | 2.371E-017 | AA-GA-GG | 1.921E-010 | AA-GA-GG | 0.000000013 | AA-GA-GG |
| rs6957 | 41830606 | untranslated-3 | AXL | 2.371E-017 | AA-GA-GG | 1.921E-010 | AA-GA-GG | 4.648E-008 | AA-GA-GG |
| rs758643 | 41991524 | unknown | CEACAM21 | 2.804E-017 | TT-TC-CC | 5.807E-011 | TT-TC-CC | 0.00003148 | TT-TC-CC |
| rs1029804 | 42107207 | unknown | LOC100505495 | 6.567E-017 | GG-GT-TT | 1.308E-015 | GG-GT-TT | 2.074E-019 | GG-GT-TT |
| rs8109167 | 41823167 | intron | AXL | 7.372E-017 | GG-TG-TT | 1.921E-010 | GG-TG-TT | 1.579E-008 | GG-TG |
| rs1046909 | 41882712 | intron | B3GNT8 | 7.383E-017 | CC-TC-TT | 0.000004939 | CC-TC-TT | 0.0000759 | CC-TC-TT |
| rs12973063 | 42009673 | unknown | CEACAM21 | 7.93E-017 | GG-GA-AA | 8.585E-016 | GG-GA-AA | 0.000002488 | GG-GA-AA |
| rs12983047 | 41834499 | unknown | AXL | 1.145E-016 | AA-GA-GG | 2.573E-009 | AA-GA-GG | 2.304E-007 | AA-GA-GG |
| rs8105161 | 41839631 | intron | AXL | 1.145E-016 | TT-CT-CC | 3.558E-009 | TT-CT-CC | 7.078E-008 | TT-CT-CC |
| rs4803464 | 41918054 | intron | B3GNT8 | 6.912E-016 | AA-AG-GG | 7.836E-007 | AA-AG-GG | 0.0000013 | AA-AG-GG |
| rs2302188 | 42085873 | missense | LOC100505495 | 1.12E-015 | AA-AG-GG | 2.221E-018 | AA-AG-GG | 8.109E-015 | AA-AG-GG |
| rs2191139 | 42001210 | unknown | CEACAM21 | 1.927E-015 | TT-TC-CC | 1.964E-008 | TT-TC-CC | 0.00008383 | TT-TC-CC |
| rs7255742 | 42049821 | unknown | LOC100505495 | 1.967E-015 | AG-AA-GG | 2.784E-013 | AA-AG-GG | 2.137E-019 | AA-AG-GG |
| rs1029804 | 42107207 | unknown | CEACAM21 | 5.78E-015 | GG-GT-TT | 6.004E-011 | GG-GT-TT | 0.00000199 | GG-GT-TT |
| rs2304234 | 41748753 | intron | AXL | 2.155E-014 | AA-GA-GG | 0.00002282 | AA-GA-GG | 0.000261 | AA-GA-GG |
| rs2241713 | 41869468 | intron | B3GNT8 | 1.314E-013 | GG-CG-CC | 0.000004495 | GG-CG-CC | 0.00004558 | GG-CG-CC |
| rs887391 | 41985624 | unknown | CEACAM21 | 3.053E-013 | CC-CT-TT | 1.605E-007 | CC-CT-TT | 0.003628 | CC-CT-TT |
| rs11083616 | 41865643 | intron | B3GNT8 | 3.437E-013 | AA-GA-GG | 0.00000466 | AA-GA-GG | 0.0000206 | AA-GA-GG |
| rs4803457 | 41861359 | intron | B3GNT8 | 3.437E-013 | CC-TC-TT | 0.00000466 | CC-TC-TT | 0.0000621 | CC-TC-TT |
| rs11666933 | 41862253 | intron | B3GNT8 | 3.809E-013 | AA-GA-GG | 0.000004297 | AA-GA-GG | 0.00007533 | AA-GA-GG |
| rs4803449 | 41734666 | intron | AXL | 2.241E-012 | CC-TC-TT | 0.001843 | CC-TC-TT | 0.0007176 | CC-TC-TT |
| rs4090570 | 42116126 | unknown | CEACAM4 | 4.026E-012 | CC-CT-TT | 0.001642 | CC-CT-TT | 0.006108 | CC-CT-TT |
| rs2191240 | 42103501 | unknown | LOC100505495 | 4.072E-012 | GG-GT-TT | 1.516E-011 | GG-GT-TT | 6.782E-019 | GG-GT-TT |
| rs2317314 | 42062307 | unknown | CEACAM21 | 4.821E-012 | CC-CT-TT | 1.07E-012 | CC-CT-TT | 0.000005914 | CC-CT-TT |
| rs2191240 | 42103501 | unknown | CEACAM21 | 5.156E-012 | GG-GT-TT | 4.594E-016 | GG-GT-TT | 1.043E-007 | GG-GT-TT |
| rs12610088 | 42020112 | unknown | CEACAM21 | 5.506E-012 | CC-CT-TT | 2.448E-009 | CC-CT-TT | 0.0003046 | CC-CT-TT |
| rs12981943 | 42019187 | unknown | CEACAM21 | 6.146E-012 | GG-GA-AA | 1.586E-009 | GG-GA-AA | 0.0003289 | GG-GA-AA |
| rs11672691 | 41985587 | unknown | CEACAM21 | 1.644E-011 | AA-AG-GG | 1.031E-007 | AA-AG-GG | 0.003572 | AA-AG-GG |
| rs16975338 | 42058025 | unknown | CEACAM21 | 1.911E-011 | AG-GG | 5.661E-014 | AG-GG | 2.774E-008 | AA-AG-GG |
| rs474481 | 40665924 | unknown | ZNF780A | 3.629E-011 | GG-AG-AA | 2.083E-012 | GG-AG-AA | 0.00001112 | GG-AG-AA |
| rs34466648 | 40433042 | coding-synon | ZNF780A | 4.876E-011 | GG-AG-AA | 0.01291 | GG-AA-AG | 0.06617 | AA-GG-AG |
| rs2231738 | 40450048 | unknown | ZNF780A | 4.932E-011 | AA-GA-GG | 0.00009891 | AA-GG-GA | 0.001084 | GG-AA-GA |
| rs3760920 | 40442558 | unknown | ZNF780A | 5.272E-011 | CC-TC-TT | 0.01144 | CC-TT-TC | 0.01882 | TT-CC-TC |
| rs3745938 | 42071167 | unknown | LOC100505495 | 5.314E-011 | CA-CC-AA | 0.00000131 | CC-CA-AA | 0.000001257 | CA-AA-CC |
| rs7247842 | 42083673 | coding-synon | LOC100505495 | 5.314E-011 | CT-CC-TT | 0.00001353 | CC-CT-TT | 0.000001257 | CT-TT-CC |
| rs740591 | 42084586 | intron | LOC100505495 | 5.314E-011 | AG-AA-GG | 0.00001353 | AA-AG-GG | 0.000001257 | AG-GG-AA |
| rs8106269 | 40447257 | unknown | ZNF780A | 6.262E-011 | CC-TC-TT | 0.0002237 | TT-CC-TC | 0.001845 | TT-CC-TC |
| rs8109718 | 40447840 | unknown | ZNF780A | 6.262E-011 | CC-TC-TT | 0.0002237 | TT-CC-TC | 0.002637 | TT-CC-TC |
| rs12462093 | 41984128 | unknown | LOC100505495 | 6.346E-011 | TC-TT-CC | 1.849E-019 | TT-TC-CC | 5.371E-014 | TT-TC-CC |
| rs6508987 | 42120778 | unknown | CEACAM4 | 7.485E-011 | AA-AG-GG | 0.0003868 | AA-AG-GG | 0.008244 | AA-AG-GG |
| rs7260507 | 41947625 | near-gene-5 | B3GNT8 | 1.389E-010 | AA-CA-CC | 0.000005541 | AA-CA-CC | 0.0002377 | AA-CA-CC |
| rs7257657 | 42082326 | near-gene-5 | LOC100505495 | 2.519E-010 | AG-GG | 0.00008155 | AG-GG | 0.001288 | AG-GG |
| rs10421785 | 42068030 | unknown | LOC100505495 | 2.901E-010 | CA-AA | 0.00002686 | CA-AA | 0.001288 | CA-AA |
| rs758350 | 42085162 | intron | LOC100505495 | 2.949E-010 | CT-TT | 0.00002686 | CT-TT | 0.0007341 | CT-TT |
| rs10421584 | 42067978 | unknown | LOC100505495 | 2.949E-010 | GA-AA | 0.00002686 | GA-AA | 0.001288 | GA-AA |
| rs2215324 | 42120283 | unknown | CEACAM4 | 2.971E-010 | AA-AG-GG | 0.001225 | AA-AG-GG | 0.009485 | AA-AG-GG |
| rs8109627 | 41822986 | intron | AXL | 3.264E-010 | TT-CT-CC | 0.00002513 | TT-CT-CC | 0.0002727 | TT-CT-CC |
| rs3760919 | 40442993 | unknown | ZNF780A | 4.313E-010 | AA-GA-GG | 0.006192 | GG-AA-GA | 0.002752 | GG-AA-GA |
| rs2241715 | 41856886 | intron | B3GNT8 | 5.767E-010 | GG-TG-TT | 0.0001956 | GG-TG-TT | 0.007973 | GG-TG-TT |
| rs2241714 | 41869392 | missense | B3GNT8 | 6.596E-010 | GG-AG-AA | 0.0001942 | GG-AG-AA | 0.007339 | GG-AG-AA |
| rs1800469 | 41860296 | ,near-gene-5 | B3GNT8 | 6.75E-010 | CC-TC-TT | 0.0001715 | CC-TC-TT | 0.009191 | CC-TC-TT |
| rs4802113 | 41740895 | intron | AXL | 7.143E-010 | TT-CT-CC | 0.004508 | TT-CT-CC | 0.01743 | TT-CT-CC |
| rs7246525 | 41737996 | intron | AXL | 2.079E-009 | AA-GA-GG | 0.0111 | AA-GA-GG | 0.009842 | AA-GA-GG |
| rs4803455 | 41851509 | intron | B3GNT8 | 2.146E-009 | AA-CA-CC | 0.0004879 | AA-AC-CC | 0.005567 | AA-CA-CC |
| rs4802111 | 41734059 | intron | AXL | 3.275E-009 | CC-TC-TT | 0.004942 | CC-TC-TT | 0.022 | CC-TC-TT |
| rs4803448 | 41734560 | intron | AXL | 5.425E-009 | TT-CT-CC | 0.006571 | TT-CT-CC | 0.01937 | TT-CT-CC |
| rs16975338 | 42058025 | unknown | LOC100505495 | 6.263E-009 | AG-GG | 3.968E-010 | AG-GG | 1.095E-015 | AA-AG-GG |
| rs10401344 | 41192417 | intron | NUMBL | 9.554E-009 | CC-TC-TT | 0.00000039 | CC-TC-TT | 3.158E-008 | CC-TC-TT |
| rs2355720 | 40440064 | intron | ZNF780A | 0.000000016 | TT-CT-CC | 0.02128 | TT-CC-CT | 0.1223 | CC-TT-CT |
| rs3786938 | 40441622 | near-gene-5 | ZNF780A | 1.607E-008 | GG-TG-TT | 0.04681 | GG-TT-TG | 0.1051 | TT-GG-TG |
| rs7249890 | 40441024 | near-gene-5 | ZNF780A | 1.619E-008 | TT-CT-CC | 0.04495 | TT-CC-CT | 0.07561 | TT-CT |
| rs2215324 | 42120283 | unknown | CEACAM21 | 2.031E-008 | AA-AG-GG | 0.00006658 | AA-AG-GG | 0.01062 | AA-AG-GG |
| rs186816 | 40594660 | intron | ZNF780A | 4.436E-008 | CC-TC-TT | 1.302E-007 | CC-TC-TT | 1.853E-007 | CC-TC |
| rs1041992 | 42132273 | coding-synon | LOC100505495 | 5.173E-008 | AG-GG | NA | NA | 0.6647 | AG-GG |
| rs6508987 | 42120778 | unknown | CEACAM21 | 5.754E-008 | AA-AG-GG | 0.00002409 | AA-AG-GG | 0.04661 | AA-AG-GG |
| rs371500 | 40480923 | intron | ZNF780A | 6.809E-008 | CC-TC-TT | 4.931E-007 | CC-TC-TT | 0.0113 | CC-TC |
| rs10402038 | 40637615 | unknown | ZNF780A | 7.343E-008 | TT-CT-CC | 7.448E-009 | TT-CT-CC | 0.0001668 | TT-CT-CC |
| rs16974093 | 40650002 | unknown | ZNF780A | 7.343E-008 | GG-AG-AA | 7.448E-009 | GG-AG-AA | 0.0002193 | GG-AG-AA |
| rs337799 | 40591182 | intron | ZNF780A | 7.343E-008 | TT-CT-CC | 1.191E-008 | TT-CT-CC | 0.0001668 | TT-CT-CC |
| rs12461895 | 41848347 | intron | B3GNT8 | 1.543E-007 | CC-AC-AA | 0.006515 | CC-AC-AA | 0.128 | CC-AC-AA |
| rs4141218 | 42081907 | near-gene-5 | LOC100505495 | 1.633E-007 | TC-TT-CC | 4.427E-011 | TT-TC-CC | 5.456E-011 | TT-TC-CC |
| rs8109718 | 40447840 | unknown | ZNF780A | 2.264E-007 | CC-TC-TT | 0.000007997 | TT-CC-TC | 0.03206 | CC-TC-TT |
| rs8106269 | 40447257 | unknown | ZNF780A | 2.264E-007 | CC-TC-TT | 0.000007997 | TT-CC-TC | 0.03561 | CC-TC-TT |
| rs3901991 | 42128922 | intron | LOC100505495 | 2.292E-007 | TC-CC | 0.2419 | TC-CC | 0.6952 | TC-CC |
| rs4803471 | 41963749 | unknown | LOC100505495 | 2.295E-007 | TT-TC-CC | 3.323E-010 | TT-TC-CC | 0.001875 | TT-TC-CC |
| rs3745205 | 40948202 | intron | SERTAD3 | 3.362E-007 | AA-AC-CC | 0.000008711 | AA-AC-CC | 0.0003802 | AA-AC-CC |
| rs929502 | 42130776 | intron | CEACAM4 | 0.000000337 | GG-GA-AA | 0.0009423 | GG-GA-AA | 0.07069 | GG-GA-AA |
| rs8111595 | 41989138 | unknown | LOC100505495 | 3.809E-007 | AC-CC | 0.01018 | AC-CC | NA | NA |
| rs890934 | 41227968 | intron | NUMBL | 4.214E-007 | TT-TG-GG | 0.00004966 | TT-TG-GG | 6.448E-009 | TT-TG-GG |
| rs421533 | 40579468 | intron | ZNF780A | 4.227E-007 | CC-TC-TT | 2.071E-008 | CC-TC-TT | 0.003179 | CC-TC |
| rs426950 | 40562386 | near-gene-5 | ZNF780A | 4.227E-007 | TT-CT-CC | 3.117E-008 | TT-CT-CC | 0.003179 | TT-CT |
| rs234346 | 40467676 | unknown | ZNF780A | 4.227E-007 | TT-CT-CC | 1.248E-007 | TT-CT-CC | 0.002509 | TT-CT |
| rs234370 | 40477465 | intron | ZNF780A | 4.227E-007 | AA-GA-GG | 1.248E-007 | AA-GA-GG | 0.003179 | AA-GA |
| rs8105066 | 40489470 | unknown | ZNF780A | 4.227E-007 | GG-AG-AA | 1.248E-007 | GG-AG-AA | 0.003179 | GG-AG |
| rs459091 | 40561175 | intron | ZNF780A | 4.361E-007 | AA-GA-GG | 1.248E-007 | AA-GA-GG | 0.003179 | AA-GA |
| rs2887292 | 40947448 | coding-synon | SERTAD3 | 5.667E-007 | GG-GA-AA | 0.000008711 | GG-GA-AA | 0.0003802 | GG-GA-AA |
| rs35314597 | 40947751 | coding-synon | SERTAD3 | 5.667E-007 | AA-AG-GG | 0.000008711 | AA-AG-GG | 0.0003802 | AA-AG-GG |
| rs10421785 | 42068030 | unknown | CEACAM21 | 6.709E-007 | CA-AA | 0.001786 | CA-AA | 0.0002254 | CA-AA |
| rs2316973 | 41953928 | unknown | LOC100505495 | 6.818E-007 | GG-GT-TT | 0.00001334 | GT-TT | 0.0004636 | GT-TT |
| rs186816 | 40594660 | intron | ZNF780A | 6.953E-007 | CC-TT-TC | 2.089E-007 | CC-TC-TT | 0.006835 | CC-TC |
| rs12462203 | 41732423 | intron | AXL | 7.337E-007 | TT-CT-CC | 0.008696 | TT-CT-CC | 0.006282 | TT-CT-CC |
| rs8110782 | 42073623 | unknown | CEACAM21 | 7.447E-007 | GG-GA-AA | 0.00000554 | GA-AA | NA | NA |
| rs10421584 | 42067978 | unknown | CEACAM21 | 7.862E-007 | GG-GA-AA | 0.001786 | GA-AA | 0.0002254 | GA-AA |
| rs758350 | 42085162 | intron | CEACAM21 | 7.862E-007 | CC-CT-TT | 0.001786 | CT-TT | 0.0002297 | CT-TT |
| rs1529717 | 41883198 | intron | B3GNT8 | 8.536E-007 | TT-TC-CC | 0.001217 | TT-TC-CC | 0.1485 | TT-TC-CC |
| rs7260605 | 41947635 | near-gene-5 | B3GNT8 | 8.549E-007 | CC-TC-TT | 0.0005006 | CC-TC-TT | 0.0001656 | CC-TC-TT |
| rs4803403 | 41412942 | unknown | CYP2G1P | 8.921E-007 | AA-GA-GG | 0.000000134 | AA-GA-GG | 0.0003037 | AA-GG-GA |
| rs4803369 | 41315980 | unknown | EGLN2 | 8.933E-007 | AA-AG-GG | 0.0006621 | AA-AG-GG | 0.003136 | AA-AG-GG |
| rs2109075 | 42152229 | unknown | CEACAM4 | 9.256E-007 | CC-TC-TT | 0.0003968 | CC-TC-TT | 0.02248 | CC-TC-TT |
| rs12462093 | 41984128 | unknown | CEACAM21 | 9.929E-007 | TT-TC-CC | 0.000001389 | TT-TC-CC | 0.01749 | TT-TC-CC |
| rs35926932 | 40952148 | near-gene-5 | SERTAD3 | 0.000001046 | AA-AG-GG | 0.000008711 | AA-AG-GG | 0.0003802 | AA-AG-GG |
| rs2561542 | 41189091 | intron | NUMBL | 0.00000111 | TT-TC-CC | 1.839E-008 | TT-TC-CC | 5.566E-013 | TT-TC-CC |
| rs170901 | 40532800 | unknown | ZNF780A | 0.000001193 | GG-AG-AA | 0.00000284 | GG-AG-AA | 0.02475 | GG-AG |
| rs2250994 | 41176403 | intron | NUMBL | 0.000001215 | AA-AG-GG | 5.523E-009 | AA-AG-GG | 5.566E-013 | AA-AG-GG |
| rs4150990 | 40930577 | intron | SERTAD3 | 0.000001352 | GG-GA-AA | 0.00001698 | GG-GA-AA | 0.0001139 | GG-GA-AA |
| rs3736329 | 41313202 | intron | EGLN2 | 0.000001387 | TT-TG-GG | 0.0006621 | TT-TG-GG | 0.002448 | TT-TG-GG |
| rs2561543 | 41187439 | intron | NUMBL | 0.000001392 | TT-TC-CC | 1.839E-008 | TT-TC-CC | 5.566E-013 | TT-TC-CC |
| rs24136 | 40596207 | intron | ZNF780A | 0.000001402 | TT-CT-CC | 0.00000222 | TT-CT-CC | 0.01855 | TT-CT |
| rs337789 | 40599603 | unknown | ZNF780A | 0.000001402 | CC-AC-AA | 0.00000222 | CC-AC-AA | 0.01855 | CC-AC |
| rs4577216 | 40569551 | unknown | ZNF780A | 0.00000142 | TT-CT-CC | 3.479E-007 | TT-CC-CT | 0.01855 | TT-CT |
| rs2005832 | 41983822 | unknown | CEACAM21 | 0.000001491 | CC-CT-TT | 1.035E-007 | CC-CT-TT | 0.002606 | CC-CT-TT |
| rs2231738 | 40450048 | unknown | ZNF780A | 0.000001555 | AA-GG-GA | 0.000003894 | AA-GG-GA | 0.04525 | AA-GA-GG |
| rs929502 | 42130776 | intron | CEACAM21 | 0.000001571 | GG-GA-AA | 0.00003991 | GG-GA-AA | 0.01771 | GG-GA-AA |
| rs2561547 | 41180687 | intron | NUMBL | 0.00000178 | CC-CT-TT | 2.884E-009 | CC-CT-TT | 5.273E-013 | CC-CT-TT |
| rs3733829 | 41310571 | intron | EGLN2 | 0.000001854 | CC-CT-TT | 0.0006621 | CC-CT-TT | 0.002448 | CC-CT-TT |
| rs2604860 | 41167721 | unknown | NUMBL | 0.000002081 | TT-TC-CC | 3.422E-008 | TT-TC-CC | 1.473E-011 | TT-TC-CC |
| rs10411347 | 41166954 | unknown | NUMBL | 0.000002081 | CC-CT-TT | 3.422E-008 | CC-CT-TT | 2.843E-011 | CC-CT-TT |
| rs170900 | 40553966 | intron | ZNF780A | 0.000002251 | GG-TG-TT | 0.001231 | GG-TG-TT | 0.0007302 | TT-GG-TG |
| rs7249286 | 40550767 | intron | ZNF780A | 0.000002251 | CC-TC-TT | 0.001397 | CC-TT-TC | 0.001803 | TT-CC-TC |
| rs7257657 | 42082326 | near-gene-5 | CEACAM21 | 0.000002795 | AG-GG | 0.009149 | AG-GG | 0.0002254 | AG-GG |
| rs3760919 | 40442993 | unknown | ZNF780A | 0.000003279 | AA-GG-GA | 0.0004094 | GG-AA-GA | 0.07389 | AA-GA-GG |

* Genotypes are ordered by mean expression values from the smaller to the higher.
